# The empirical characteristics of human pattern vision defy theoretically-driven expectations

**DOI:** 10.1371/journal.pcbi.1006585

**Published:** 2018-12-04

**Authors:** Peter Neri

**Affiliations:** Laboratoire des Systèmes Perceptifs, Département d’études cognitives, École normale supérieure, PSL University, CNRS, 75005 Paris, France; University of California at Berkeley, UNITED STATES

## Abstract

Contrast is the most fundamental property of images. Consequently, any comprehensive model of biological vision must incorporate this attribute and provide a veritable description of its impact on visual perception. Current theoretical and computational models predict that vision should modify its characteristics at low contrast: for example, it should become broader (more lowpass) to protect from noise, as often demonstrated by individual neurons. We find that the opposite is true for human discrimination of elementary image elements: vision becomes sharper, not broader, as contrast approaches threshold levels. Furthermore, it suffers from increased internal variability at low contrast and it transitions from a surprisingly linear regime at high contrast to a markedly nonlinear processing mode in the low-contrast range. These characteristics are hard-wired in that they happen on a single trial without memory or expectation. Overall, the empirical results urge caution when attempting to interpret human vision from the standpoint of optimality and related theoretical constructs. Direct measurements of this phenomenon indicate that the actual constraints derive from intrinsic architectural features, such as the co-existence of complex-cell-like and simple-cell-like components. Small circuits built around these elements can indeed account for the empirical results, but do not appear to operate in a manner that conforms to optimality even approximately. More generally, our results provide a compelling demonstration of how far we still are from securing an adequate computational account of the most basic operations carried out by human vision.

## Author summary

We can view cortex from two fundamentally different perspectives: a powerful device for performing optimal inference, or an assembly of biological components not built for achieving statistical optimality. The former approach is attractive thanks to its elegance and potentially wide applicability, however the basic facts of human pattern vision do not support it. Instead, they indicate that the idiosyncratic behaviour produced by visual cortex is primarily dictated by its hardware components. The output of these components can be steered towards optimality by our cognitive apparatus, but only to a marginal extent. We conclude that current theories of visually-guided behaviour are at best inadequate, calling for a rebalanced view of the roles played by theoretical and experimental thinking about this function.

## Introduction

The most essential property of images is contrast: without contrast, there is no image; without image, there is no vision. This is not to say that vision is solely about perceiving contrast: image information is perceptually re-formatted into numerous perceptual descriptors (e.g. motion, disparity, gloss) that afford greater richness than mere contrast maps. Nevertheless, this richer representation still relies on a primitive image defined by contrast [1, 2], without which no subsequent processing can take place. Perhaps as a consequence of this simple fact, contrast has been by far the most manipulated feature in vision science [3, 4]. It is uncontroversial to say that the facts of contrast perception are the basic facts of human vision, and that any candidate model of visual processing cannot be satisfactory unless it incorporates and predicts the effect of contrast.

There are many such models. Coarsely speaking, they range between those constrained by statistically optimal objectives on the one hand [5], and those constrained by cortical circuitry on the other [6, 7]. Although these two formulations are not mutually exclusive and may co-exist under some conditions [8–10], they represent fundamentally different ways of thinking about sensory processing. In the approach driven by optimality, the primary focus is on a cost function associated with the task at hand (e.g. incorrect identification of the target signal). Behaviour is then parameterized to minimize costs, and this parameterization is projected onto expected values for the empirically measured quantities.

In the approach constrained by what we currently know about cortical circuitry [11], optimality is never explicitly represented nor prioritized. The primary focus is on identifying the simplest circuit that is consistent with the empirical observations [6, 7]. Whether the identified circuit is optimal or not is, to a large extent, besides the point. This class of models lacks the elegance that comes with optimality principles, however it is justified by the notion that sensory systems have evolved different building blocks along a tortuous path where, at any given time, the critical factor has been to perform well enough to survive, without the need to represent and/or achieve statistical optimality [12, 13]. The system may find itself bound up with components that may be sub-optimal under a number of relevant situations. The only option is to make do with whatever hardware is available.

Another important distinction sets apart mechanistic from non-mechanistic models. Here, a mechanistic model is one that acts directly on the input stimulus (e.g. 2D image) and computes a behavioural response through a pre-specified fully parameterized algorithm [6, 14]. When a stimulus property is changed (e.g. image contrast), the model may behave differently but only as a consequence of its internal computations. Gain control implemented via fixed-parameter divisive normalization is an example of this class of models [15]. Non-mechanistic models, on the other hand, bypass the issue of what exact mechanism determines their change in behaviour. It may be hypothesized, for example, that at high contrast the system applies different read-out rules than at low contrast (e.g. [16, 17]), without implementing the change via an explicit image-based computation of contrast.

Our results speak in favour of mechanistic circuit-based models, and are largely *opposite* to the predictions associated with optimal inference. We propose a fully specified mechanistic model based on a combination of simple-like and complex-like units, where the balance between the two is dictated by contrast via divisive gain control. Although this architecture is not definitive and alternative accounts may be equally effective, it does indicate that the critical factors shaping sensory discrimination may be primarily determined by the kind of hardware that is available to carry out the computation, as opposed to theoretical optimization of the computation itself. To some extent, the hardware must be informed by what is optimal in the first place, but our data suggest that this connection involves a balancing act, rather than optimality driving implementation as may be conceived from a primarily theoretical perspective [5, 18, 19].

## Methods

### Ethics statement

Approved by departmental IRB committee protocol ID# LSP0027. All participants gave explicit written consent.

### Stimuli, task, procedure

The overarching goal of the experimental manipulations adopted for this study is to obtain a detailed characterization of how the human visual process represents simple image elements [1, 2], more specifically a vertical segment. To this end, we designed simple detection/discrimination tasks involving briefly flashed bar-like elements [20, 21] (see detailed description below). In order to gain greater insight into the underlying sensory mechanisms, we superimposed a noise mask onto the target bar and studied how the detailed structure of the mask impacted perceptual judgments [22–24].

Stimuli consisted of 13 adjacent vertical bars measuring ∼0.15×2 deg (width×height). The different bars took luminance values which we record as vector **s**. For example, *s*_1_ specifies the luminance of the first bar in the stimulus array, *s*_2_ the luminance of the second bar immediately adjacent to the first bar, and so on for the remaining 11 elements of **s**. Each trial contained two such stimuli: the ‘non-target’ stimulus **s**^[0]^ and the ‘target’ stimulus **s**^[1]^. Below we adopt the convention of referring to both stimuli using **s**^[*q*]^ where *q* may denote either target (*q* = 1) or non-target (*q* = 0). Each stimulus lasted 50 ms and was constructed by summing two components: a ‘noise’ component denoted by vector **n** and a ‘signal’ component denoted by vector **t**, so that **s**^[*q*]^ = **n**^[*q*]^ + **t**^[*q*]^. Both **n** and **t** differ for target and non-target stimuli (hence the superscript *q*), however the difference is due to statistical sampling in the case of **n**, while it is deterministic in the case of **t**: **t**^[0]^ and **t**^[1]^ are pre-specified differently at the start of each experiment (see further below for details) and remain unchanged throughout the experiment, while **n**^[0]^ and **n**^[1]^ are different samples from the same noise generator and differ not only between themselves, but also from one trial to the next (except for double-pass protocols, to be detailed in relation to the measurement of internal noise). In all experiments, each element of **n** was independently and randomly drawn from a zero-mean Gaussian distribution with SD *σ*_N_, and **t** was zero everywhere except for the central element (corresponding to the 7^th^ bar in the stimulus) which took value *ρ*^[*q*]^ (target signal **t**^[1]^ and non-target signal **t**^[0]^ only differed as specified by the two scalar quantities *ρ*^[1]^ and *ρ*^[0]^). The addition of target/non-target stimulus **t** therefore involved a simple luminance offset (*ρ*) applied to the central bar of noise stimulus **n**. The different tasks involved detecting/discriminating offsets of this kind.

In the ‘bright-bar detection’ experiments, the target stimulus contained a central bright bar of intensity *ρ*^[1]^ > 0 added onto the noise (below we specify the exact values that *ρ* could take), while the non-target stimulus was just noise (*ρ*^[0]^ = 0). In the ‘dark-bar detection’ experiments *ρ*^[1]^ < 0 (target bar was dark rather than bright, i.e. it involved a luminance decrement rather than an increment applied to the central bar in the stimulus). In all experiments, the spatial location of the central bar was marked by two red vertical bars immediately above and below the stimulus measuring the same size as the stimulus bars; these two markers served to minimize spatial uncertainty [20] and remained on the screen throughout the block. In all experiments bar ‘foveal detection’, target and non-target stimuli were simultaneously presented at an eccentricity of 2.5 deg on opposite sides of a fixation cross measuring ∼0.5×0.5 deg, each flanked above and below by the red markers (for a total of 4 markers displayed continuously). In the ‘foveal detection’ experiments, target and non-target stimuli were presented in temporal succession (separated by a 500-ms gap) at fixation marked by a smaller cross (∼9×9 arcmin), flanked by two red markers above and below (we reduced the size of the fixation cross to minimize overlap with the stimulus). In the ‘mixed-polarity detection’ experiment (also termed ‘energy extraction’ task in Results), *ρ*^[1]^ could be either positive or negative on any given trial by random assignment. In the ‘polarity discrimination’ experiments, *ρ*^[1]^ > 0 and *ρ*^[0]^ = −*ρ*^[1]^: the target stimulus contained a central bright bar while the non-target stimulus contained a central dark bar of opposite intensity.

*ρ*^[1]^ and *σ*_N_ values were randomly assigned at the beginning of each 50-trial block and were kept constant throughout the block. *ρ*^[1]^ could take one of 4 integer values between 1 and 4 when expressed as multiple of *σ*_N_; the assigned value is the stimulus signal-to-noise ratio (SNR). *σ*_N_ was a multiple *c* of physical monitor luminance 0.3 cd/m^2^ against fixed background luminance of 30 cd/m^2^, where *c* could be one of 4 logarithmically spaced values 1, 2, 4 or 8 (roughly equivalent to same figures as % contrast); the assigned value is the stimulus contrast level. For example, if SNR level was set to 2 and contrast level was set to 4 in the bright-bar detection experiment, actual stimuli on the monitor were specified as follows: the non-target stimulus consisted of 13 bars, each taking a random value from a zero-mean Gaussian distribution with SD 4×0.3 cd/m^2^ (see above definition for *σ*_N_) added onto 30 cd/m^2^ (background); the target stimulus consisted of 13 bars, each taking a random value from the same distribution, with a luminance offset added onto the middle bar of 2×4×0.3 cd/m^2^ (see above definition for *ρ*).

At the end of each trial, observers were asked to indicate which stimulus contained the target (left-vs-right location for all experiments except foveal detection where they reported first-vs-second interval); immediately after their response, they received trial-by-trial feedback (correct/incorrect) and triggered the next stimulus presentation with a random delay of 500-700 ms. At the end of each block, they also received a summary of their overall performance (% correct) on their last block and across all blocks collected to that point. For each different task, observers were explicitly informed of target and non-target characteristics and were shown noiseless versions of these stimuli during an initial set of practice trials, so as to eliminate any potential ambiguity regarding the specification of target and non-target stimuli.

### Observers and data mass

We collected a total of 614050 trials. Eight observers participated in the peripheral bright-bar detection experiments for a total of 124825 trials (>15K trials per observer), and an additional 12825 double-pass trials (see section below). For the dark-bar detection experiments: 4 observers, 100075 trials (>25K per observer), 12525 double-pass trials. Foveal bright-bar detection experiments: 8 observers, 116150 trials (>14K trials per observer), 12600 double-pass trials. Polarity-discrimination experiments: 8 observers, 109725 (∼14K trials per observer), 14725 double-pass trials. Mixed-polarity detection experiments: 9 observers, 97825 trials (∼11K trials per observer), 12775 double-pass trials. All observers were naïve to the purpose of the experiments and were paid 9 euros/hour.

### Internal noise estimation

We estimated internal noise using established double-pass techniques [25, 26], arguably the most effective psychophysical tool for tackling this problem [27]. Blocks consisted of 50 trials for which the second 25 trials (trial #26 to #50) contained the same stimuli that were presented during the first 25 trials (trial #1 to #25) in randomly permuted trial order [28]. *p*_a_ is the fraction of paired trials on which observers generated the same response; *p*_c_ is the overall fraction of correct responses. These two measurements are sufficient to constrain an unbiased 2-parameter signal detection theory (SDT) model with output *u*(*d*_ext_ + *ϵ*_int_) where *u*(.) is the Heaviside function producing a binary response, *d*_ext_ is a random variable from a normal distribution with mean ${\text{d}}_{\text{in}}^{\prime }$ (free parameter) and *ϵ*_int_ is a random variable from a zero-mean Gaussian distribution with SD *N*_int_ (free parameter). *d*_ext_ is entirely stimulus-dependent so that, when the same stimulus is repeated, its value remains unchanged. *ϵ*_int_ is instead fully decoupled from the stimulus: it is randomly assigned anew on every iteration. Internal noise is defined as the ratio between the standard deviation (SD) of the decision variable driven by internal noise fluctuations (*ϵ*_int_), and the standard deviation of the decision variable driven by external noise fluctuations (*d*_ext_). In SDT, it is customary to set the SD of the latter source to 1, effectively serving as unit in perceptual space [29] (this space, by its very nature, does not possess a native unit of measurement like spikes/sec for neurons); in such units, *N*_int_ is therefore the internal noise value itself [25]. The two parameters ${\text{d}}_{\text{in}}^{\prime }$ and *N*_int_ are estimated by simply minimizing the mean square error between the empirically measured {*p*_c_, *p*_a_} values and the corresponding values generated by the SDT model [26]. The quantity ${\text{d}}_{\text{in}}^{\prime }$ is related to, but not the same as, the standard discriminability index d′: the latter incorporates the effect of internal noise, while the former reflects discriminability within the perceptual mechanism *before* the addition of internal noise [22, 25, 26].

The two fundamental assumptions underlying the double-pass approach are: 1) the binary psychophysical decision is driven by the difference rule; 2) internal noise is late and additive. The first assumption implies that the sensory circuitry engaged by the observer produces two figures of merit, one for each stimulus (or equivalently interval); the observer then chooses the interval associated with the largest figure of merit [29]. This rule is often referred to as ‘difference rule’ because it is equivalent to taking the difference between the two figures of merit (quantity submitted to *u*(.) above), and choosing one interval if the difference is >0, or the other interval if the difference is ≤0 (Heaviside function *u*(.)). The term ‘figure of merit’ refers to the likelihood that the associated stimulus contains the target signal to be detected, where ‘likelihood’ is a general term that reflects how much weight the observer places on a given stimulus, regardless of how said weight is computed (i.e. it is not intended here in the sense of Bayesian inference or other specific concepts). This construct is closely related to the notion of ‘decision variable’ [30]. No specific assumption is made as to *how* the figure of merit or decision variable is computed; in this sense, the methodology detailed here applies to any perceptual system (linear or nonlinear) provided its final output is a figure of merit (i.e. scalar [24]). There is good experimental evidence to support the difference rule, at least for low-level perceptual tasks [31, 32]. The second assumption is stronger, but it should not be interpreted literally: it neither implies that intrinsic noise originates from only one source, nor that the physiological action of said source is additive. What is assumed is that the collective action of all noise sources affecting the observer can be adequately modelled (within experimentally measurable margin) using a sizeable late additive noise source; there is good experimental evidence to support this assumption [26]. We study departures from this noise specification with computational modelling (see further below in Methods and S3 Fig).

It may appear that other important assumptions are implicitly built into the model, notably the choice of Gaussian characteristics. This choice of distribution, although common [25, 29], is arbitrary and represents a poor approximation to the perceptual mechanism [31]. However, it is largely uninfluential for the purpose of measuring the overall intensity of the noise source (whatever its distribution may be). More specifically, we have verified that other plausible distributions (e.g. Laplacian [31]) lead to estimates that only differ by amounts that cannot be resolved empirically under realistic laboratory conditions [28]. Furthermore, our focus here is *not* on absolute values but on *trends*, i.e. relative comparisons (e.g. low versus high contrast); different distributions lead to equivalent trends (provided the chosen distribution is plausible [24] and, whatever that distribution may be, it is not assumed to change between conditions).

The SDT model detailed above incorporates an additional hidden assumption, namely that observers do not present response bias: under this assumption, the binary choice between interval 1 and interval 2 is only based on a comparison between the two figures of merit generated by the perceptual mechanism, without any stimulus-decoupled preference for either interval. In the context of our experiments, we therefore assume that observers are not biased towards reporting the left side of the screen more often than the right side (or the other way around). We have verified that, whatever bias may be present, it does not impact our conclusions. First, across our dataset bias is roughly symmetrically distributed around 0 with a small average value of -0.03 against a SD of 0.17 (S1A Fig). Second, when we recompute internal noise using a model that incorporates bias (please refer to [33] for details), the resulting estimates within the plausible range [26] (>1/5 and <5) are well correlated (coefficient of ∼0.8) with those obtained when bias is not taken into account (S1B Fig). Third, when we use the recomputed values to evaluate potential trends as a function of contrast and SNR, we find similar effects and all our conclusions remain unchanged. In the remaining part of this article we have chosen to report estimates without correction for bias because they tend to be more robust (the underlying SDT framework presents less degrees of freedom).

### Separability index

We construct a 4×4 matrix (denoted by uppercase letter **S**) for a given scalar quantity (e.g. internal noise) specifying the value of that quantity for each possible pairing of contrast and SNR. For example, element *S*_*ij*_ of matrix **S** may specify the intensity of internal noise (or any other chosen metric) for SNR level *i* and contrast level *j* (the 4 possible levels of SNR and contrast are specified above). We adopt two quantitative estimators of separability for **S**, where ‘separable’ is intended in the usual sense [34] that **S** reflects the product of one-dimensional tuning functions separately defined along the two dimensions of contrast and SNR: if **x** is the vector that specifies tuning as a function of SNR, and **y** is the vector that specifies tuning as a function of contrast, then **S** = **x** ⊗ **y** where ⊗ is outer product (*S*_*ij*_ = *x*_*i*_*y*_*j*_). The first approach involves computing the separable prediction ${\text{S}}^{*}:\;S_{ij}^{*}=\sum_{j=1}^{4}S_{ij}\times \sum_{i=1}^{4}S_{ij}/\sum_{i,j=1}^{4}S_{ij}$ (normalized outer product of marginals). It is easy to verify that, if **S** is separable, then S* = **S**. We therefore compare S* (prediction) with **S** (data) to estimate the degree of separability. The second approach involves computing singular values for **S**; under separability, the first singular value *v*_1_ is larger than all others [35]. We therefore compute a separability index (which we term SVD index) as $v_{1}^{2}/\sum_{i}v_{i}^{2}$ (values are squared for consistency with previous formulations [35, 36]). This index ranges between near 0 for a fully inseparable surface and 1 for a fully separable surface (for which all values except *v*_1_ are 0).

### Estimation of first- and second-order filter descriptors

We characterize the tuning characteristics of the human sensory process using a psychophysical variant of reverse correlation termed noise image classification [22–24]. The resulting descriptors can be computed directly from data in the absence of any specific assumption about the properties of the perceptual mechanism [37]. Subsequent interpretation of these empirically-derived descriptors is often assisted by the assumption that the perceptual mechanism conforms to a linear-nonlinear (LN) cascade (also termed ‘template matching’ by some literature [38], see below for definition), however we do not make that assumption here. Rather, we assess the applicability of the LN cascade and replace this model with different architectures when it is unsupported by data [14].

Before proceeding further, we define the LN cascade for application to psychophysics. As explained above, we do not commit to this model in the present study, however we often use it for normative purposes (e.g. definition of ideal observer [19] or reference point of departure for nonlinearity indices [14]). If we consider the 2AFC protocol used here, the output of a linear-nonlinear cascade is defined by the *linear* application of template **f** to the difference between target and non-target stimuli in the form 〈**f**, **s**^[1]^ − **s**^[0]^〉 (hence the term template-matching), followed by the *nonlinear* application of a decisional transducer to generate a binary correct/incorrect choice (e.g. *u*(.) above). In this notation, 〈〉 is inner product: 〈**x**, **y**〉 = ∑_*i*_ *x*_*i*_*y*_*i*_ (when adopted with matrices, Frobenius inner product is intended: 〈**X**, **Y**〉 = ∑_*ij*_ *X*_*ij*_*Y*_*ij*_).

Below we describe the procedure by which different descriptors are computed. We use the term ‘descriptor’, rather than ‘kernel’ or other similar terms more commonly adopted in the literature [24, 39], to emphasize the distinction between *describing* the data on the one hand, and *identifying* structural components of the perceptual system on the other hand [37, 40, 41]. The latter procedure *does* rely on specific assumptions about system structure, however it can be framed within the general framework of Wiener/Volterra expansion [24, 42–44] (to be outlined in the next section). In this study we adopt a combination of data-driven and model-driven approaches, depending on the problem at hand [14]. For example, we use data-driven indices of sharpness and linearity, but we then interpret their contrast-dependence via fully specified mechanistic models.

At the end of each trial, observers are asked to classify the two presented stimuli as one being target, and the other one being non-target (single binary choice: target-on-the-right/non-target-on-the-left versus target-on-the-left/non-target-on-the-right). Following stimulus classification by observers, each noise sample can therefore be indexed as **n**^[*q*,*z*]^ where *q* is 0 for non-target and 1 for target (same notation as above), while *z* is 0 for incorrect response and 1 for correct response. For example, if on a given trial the observer responds incorrectly (*z* = 0), the two noise samples presented on that trial are denoted **n**^[0,0]^ (sample presented in the non-target interval, i.e. *q* = 0) and **n**^[1,0]^ (sample presented in the target interval, i.e. *q* = 1). Classified noise samples can be exploited to derive images of the perceptual filtering mechanism engaged by human observers [23]. We derive two main descriptors of the filtering mechanism: the ‘first-order’ descriptor **h**, obtained by averaging noise samples [22]; and the ‘second-order’ descriptor **H**, derived from the covariance of noise samples [24].

The first-order descriptor consists of two sub-components: the *target-present* first-order descriptor **h**^[1]^ = *μ*(**n**^[1,1]^) − *μ*(**n**^[1,0]^) where *μ* is average across trials of the indexed type; and the *target-absent* descriptor **h**^[0]^ = *μ*(**n**^[0,0]^) − *μ*(**n**^[0,1]^). The terms ‘target-present’ and ‘target-absent’ refer to the two classes of noise samples that are exploited to derive the corresponding components: ‘target-present’ noise samples are those presented in the target interval (i.e. those added to the target signal), while ‘target-absent’ noise samples are those presented in the non-target interval (i.e. added to the non-target). As indicated by the expressions above, however, the target signal itself is never used to compute the perceptual descriptors. For example, *μ*(**n**^[1,1]^) is the average of all noise samples that were added to the target signal on trials for which the observer responded correctly. The full first-order descriptor is simply **h** = **h**^[1]^ + **h**^[0]^ or, equivalently, **h** = (2*δ*_*q*,*z*_ − 1)∑_*q*,*z*_ *μ*(**n**^[*q*,*z*]^) where *δ*_*q*,*z*_ is Kronecker *δ* (= 0 if *q* ≠ *z*, = 1 if *q* = *z*). Using similar notation, the second-order descriptor is **H** = (2*δ*_*q*,*z*_ − 1)∑_*q*,*z*_cov(**n**^[*q*,*z*]^) (same as **h** with *μ* replaced by covariance matrix cov).

We also compute first-order descriptors from noise power across spatial frequency (rather than intensity modulation across space). For this purpose, we adopt the same combination rule detailed above for **h**, except we replace each individual noise sample **n** with its Fourier power profile [45]. Although power is a positive quantity, descriptor amplitude may be negative because it is obtained by adding and subtracting average power profiles for different sets of classified noise samples (see expressions above).

### Eigen decomposition of second-order descriptors

Due to their larger dimensionality, second-order descriptors (2D) are not directly comparable to first-order descriptors (1D), and some metrics adopted in this study (notably sharpness) are only defined for 1D descriptors. It is however possible to reduce the dimensionality of second-order descriptors via eigen decomposition; the resulting eigenvectors are directly comparable to first-order descriptors. Furthermore, under a general linear/nonlinear transduction model, eigenvectors reflect the properties of front-end convolutional layers [37, 39]. More specifically, if $f_{+}^{n}$ is the *n*^th^ eigenvector associated with a positive eigenvalue, and $f_{-}^{n}$ is the *n*^th^ eigenvector associated with a negative eigenvalue, these descriptors may be viewed as filter components of a model with decision variable $r=\langle f,s\rangle +\sum_{n=1}^{N^{+}}{\langle f_{+}^{n},s\rangle }^{2}-\sum_{n=1}^{N^{-}}{\langle f_{-}^{n},s\rangle }^{2}$ where **f** is the linear filter inside the model and *N*^+^/*N*^−^ are the numbers of statistically significant positive/negative eigenvalues (see below for how eigenvalue significance was assessed).

In the expression for *r* above, the second-order eigenvectors (also termed principal dynamic modes in the engineering literature [46]) initially act as linear filters on the input stimulus **s** (similar to **f**), except their output is subjected to a squaring nonlinearity before being added/subtracted to the final response. This formulation is connected with quadratic modelling [47] and naturally incorporates common computational tools such as the energy model [48, 49]. In the context of this approach, the distinction between data description and system identification (detailed above in relation to the estimation of first-/second-order descritors) is particularly important [37]. The estimation procedure can be carried out regardless of whether the underlying perceptual system conforms to the above expression for *r*. In principle, eigenvectors may also be computed regardless; however, if they are to be interpreted as filtering components of the model implied by *r*, then of course this interpretation comes with the assumption that *r* above applies, together with an additional assumption (see below).

The connection between second-order descriptors (with associated eigenvectors) and the quadratic model for *r* above becomes evident when we recast system output via Volterra expansion [39, 43, 44, 46]. The second-order Volterra expansion of the psychophysical decision variable can be written as *r* = 〈**l**, **s**〉 + 〈**L**, **s** ⊗ **s**〉 [24] where **l** is the first-order Volterra kernel and **L** is the second-order kernel. This expression extends the concept of Taylor expansion for univariate functions to multivariate functions [50]. Only terms up to second-order are included, which may seem an overly conservative approximation; practically speaking, however, higher-order terms cannot be estimated reliably [24, 39, 44, 49], rendering the issue of little experimental relevance. When the Volterra expansion above for *r* is equated with the quadratic expression from the previous paragraph, the connection becomes evident (rewrite **L** as **B** × Δ × **B**′ where **B** is the matrix specifying the eigenvectors and Δ (diagonal) the eigenvalues; see [39]).

We estimate the second-order Volterra kernel **L** via the psychophysical second-order descriptor **H** (it is of course from the latter that we extract eigenvectors). We must assume that **H** is an acceptable approximation to **L** [51, 52]; the applicability of this notion to the behavioural context is complicated by a number of factors [44], in particular the nearly inevitable presence of a target signal (**t** in our notation above) and the decisional transducer [24] (*u*(.) in our notation above). Notwithstanding these potential sources of artefactual modulations, the correspondence between **H** and **L** is generally robust [24] and facilitated by the conditions of our experiments (e.g. lack of decisional bias, see S1A Fig).

We alert readers to the above issues without further consideration of the associated details (these issues have been addressed in prior work [21, 24, 44, 53] and the above methodology shares elements with established approaches e.g. spike-triggered covariance [46, 49]). It is relevant here that we exploit second-order descriptors to demonstrate an invariant property that is contrast *independent*, rather than contrast dependent. If the property we consider (sharpness of eigenvectors) were impacted by contrast, potential concerns about the applicability of the assumption detailed above would be of substantial concern, due to the possibility that contrast dependence may be artefactually generated by estimation issues associated with second-order descriptors [21, 24]. However we demonstrate that this property is *invariant* to contrast, despite presenting other contrast-dependent properties (e.g. overall amplitude of the descriptor), largely mitigating these potential concerns.

To determine which eigenvalues should be retained and which discarded, we compute eigenvalues from decoupled processes where individual pairs of noise samples presented on a given trial are randomly assigned to individual psychophysical responses from other trials (this approach leaves the overall statistical structure of both noise samples and psychophysical responses unaffected when considered in isolation, however it eliminates potential statistical inter-dependencies between the two). The spread of the resulting distribution defines a non-significant region for the eigenvalues estimated from the actual (coupled) perceptual process.

### Drive of perceptual tuning descriptors

This metric is designed to reflect the amount of structured modulation within a given descriptor (see above for how this object is computed), where ‘structured modulation’ refers to modulation not attributable to measurement noise. It is not intended to capture specific shape features, such as sharpness (see below). The overall modulation content of descriptor **h**/**H** is captured by scalar metric λ, the log-ratio between the descriptor root-mean-square (RMS) amplitude and the expected RMS of the corresponding descriptor generated by a decoupled process (one for which the psychophysical response bears no relation to the input stimulus, see above): $\lambda_{d}=\text{log}\left({\text{RMS}}_{d}/{\text{RMS}}_{d}^{*}\right)$ where *d* is descriptor order (*d* = 1 or 2 for first- or second-order).

The expected value of ${\text{RMS}}_{d}^{*}$ can be written out explicitly as $d\times k\times \sigma_{\text{N}}^{d}$ where $k=\sqrt{\frac{2}{m\left(1-p_{\text{c}}\right)p_{\text{c}}}}$ and *m* is the total number of collected trials [24, 54]. For a decoupled process, the expected value of λ_*d*_ is 0. Because λ is so defined with respect to a decoupled process, and because a decoupled process corresponds to 0 perceptual drive, we use the term ‘drive’ when referring to λ. For the second-order descriptor (*d* = 2), the expression above for ${\text{RMS}}_{2}^{*}$ applies to the diagonal (variance) region of the descriptor. Because the expected value for the off-diagonal region is scaled down by $\sqrt{2}$, we first multiply the off-diagonal region by this factor before computing its RMS value and the associated λ_2_. For target-present and target-absent first-order descriptors, ${\text{RMS}}_{1}^{*}$ is also scaled down by $\sqrt{2}$ compared to the full descriptor; corresponding estimates of $\lambda_{1}^{\left[q\right]}$ (*q* = 1 for target-present and 0 for target-absent) are adjusted accordingly.

### Sharpness index

Sharpness of 1D profile **h** is estimated via spectral centroid, a robust validated metric [55] intended to capture the degree of tuning displayed by first-order descriptors. We compute the one-sided power spectrum **p** of **h** across frequency axis **x**. We normalize **p** to unit sum and compute 〈**p**, **x**〉. This metric suffers from the potential confound that **h** consisting of noise alone may shift the spectral centroid to relatively high values, so that a sharpness increase may be artefactually caused by noisier estimates, rather than genuine sharpening. In our study, we can exclude this artefactual explanation not only based on visual inspection of first-order descriptors, but also based on the result that sharpness of eigenvectors from second-order descriptors is independent of contrast (see above); under the artefactual explanation, a similar contrast-dependence should be observed for second-order as well as first-order estimates.

### Nonlinearity index

We combine two established markers of nonlinear processing into one aggregate linearity index, where ‘nonlinear’ refers to departure from the template matcher model (see above for definition of this model). Our goal below is to design metrics for the two markers that return ≤0 for a linear template, and values > 0 for a system that departs from the linear template. Furthermore, the metric value should ideally scale with the overall extent of nonlinear behaviour exhibited by the system, although the latter goal is not easily achieved due to the lack of unique definition for ‘nonlinear processing’: when comparing two different nonlinear architectures, it is often difficult to identify a unique criterion by which one is viewed as being more or less nonlinear than the other.

We adopt aggregate indices of linearity/nonlinearity for the purpose of computing their contrast/SNR dependence (see below), but we do not rely solely on the indices themselves to study linear/nonlinear transitions because they represent drastic attempts at data reduction, leaving room for potential misinterpretation. In particular, the indices are derived from the drive metric (λ) defined above (see below for details). Drive itself is a coarse summary of filter descriptors; further elaboration of this metric into an aggregate linearity index carries potential pitfalls (discussed below). For this reason, we inspect individual drive values and study their contrast/SNR dependence in detail throughout this study, to ensure that the inevitable need for data reduction/visualization does not incur undesirable costs.

The first marker of nonlinear processing reflects the difference between target-present and target-absent descriptors [14, 45]. Using the λ metric defined above, we simply compute λ^[1]^ − λ^[0]^. When the two descriptors match as predicted by the template matcher model [22, 23], this metric is expected to return 0. The template matcher predicts that the two descriptors should be the same because the template is applied linearly to the stimulus (in the form of a weighted sum across stimulus elements [23], see above). For a linear template, the response to noise+target is equal to the response to noise plus the response to target [22] (using the notation adopted earlier 〈**f**, **s**^[1]^〉 = 〈**f**, **t**^[1]^ + **n**^[1]^〉 = 〈**f**, **t**^[1]^〉 + 〈**f**, **n**^[1]^〉); the additive term corresponding to the target response is independent of the noise stimulus (it only depends on the target stimulus which remains the same from trial to trial), bearing no impact on filter descriptors computed from noise samples [44], whether the samples are associated with the target (target-present) or not (target-absent). For a system that *departs* from the linear template matcher model, however, the response to noise+target cannot be decomposed into separate responses to noise and target [44] (i.e. it does not obey the superposition principle), introducing potential differences between target-present and target-absent descriptors [24]. In general, these differences produce greater modulation within target-present descriptors [24], resulting in λ^[1]^ > λ^[0]^ (positive value for the nonlinear marker).

The second marker reflects structure within the second-order descriptor against structure within the first-order descriptor: λ_2_ − λ_1_. There is a potential pitfall with this expression, in that it may incorrectly assess descriptors generated by the template matcher model (our normative model for assessing linearity). For this model, the expected value of λ_2_ is 0 [24], while the expected value of λ_1_ depends on internal noise [22]. If a given manipulation affects internal noise, it will also result in systematic variations of the above-defined marker, incorrectly indicating that the system is departing from the template matcher model when in fact this model (combined with a variation of internal noise) may explain the trend. The marker definition above is only sensible if λ_2_ is expected to differ from 0; under such conditions, internal noise will in general impact both λ_1_ and λ_2_, stabilizing the marker against internal noise variations.

To rectify this issue, we adopt the slightly modified expression (λ_1_ − λ_2_) × sign(λ_2_). For λ_2_ estimates with expected value of 0 as predicted by the template matcher, this marker will also return (on average) a value not different than 0. The marker as defined above suffers from other potential pitfalls, in particular it does not index linearity in monotonic fashion: a highly nonlinear system returns a positive value; a highly linear system with a residual nonlinear component (λ_2_ > 0) returns negative values; a fully linear system returns 0 (rather than even more negative values). In practice, however, we find that these issues do not have any substantial impact on our empirical estimates of the dependence on contrast and SNR (defined below), while at the same time aiding correct interpretation of linearity trends associated with synthetic estimates from model simulations (e.g. the modified marker correctly supports rejection of template matcher models).

The *aggregate* nonlinearity index is simply the sum of the two markers just defined (more positive index corresponds to greater nonlinearity i.e. departure from template matching; 0 or more negative index corresponds to greater linearity i.e. conformity with template matching). This index was used to compute correlation coefficients for contrast/SNR dependence of linearity. For reasons of ease of exposition, in this section we describe the adopted index as ‘nonlinearity index’, however in the article we present contrast/SNR dependence of the ‘linearity index’ by inverting its sign.

### Contrast/SNR dependence

To obtain a compact measure of whether a given metric is modulated by SNR and/or contrast, we compute the correlation coefficient between the metric values and the corresponding SNR values (to obtain SNR dependence) or contrast values (to obtain contrast dependence). This procedure is carried out separately for each observer and each experiment the observer participated in. For example, if a given observer participated in the bright-bar detection task, we measure 16 independent estimates of sharpness, each associated with a specific choice of SNR and contrast. We then compute the correlation coefficient between those 16 estimates and the corresponding SNR values to obtain the SNR dependence of the sharpness metric (similarly for contrast dependence). In some instances, we compute dependence on SNR (alternatively contrast) separately for each level of contrast (alternatively SNR). In this version of the analysis, we obtain 4 estimates of SNR (or contrast) dependence for each observer in each task.

### Ideal observer and absolute efficiency

The ideal observer for all tasks except mixed-polarity (energy extraction) belongs to the template-matching family (defined earlier) with template **f** = **t**^[1]^ − **t**^[0]^ [29]: the response *r*^[*q*]^ to target/non-target stimulus **s**^[*q*]^ is *r*^[*q*]^ = 〈**t**^[1]^ − **t**^[0]^, **s**^[*q*]^〉; the observer responds correctly if *r* = *r*^[1]^ − *r*^[0]^ is >0 (response to target stimulus greater than response to non-target stimulus), incorrectly otherwise. For the bar detection task (whether the bar is bright or dark and whether it is presented in fovea or periphery), the ideal template is zero everywhere except at the central location where the target bar is presented; at that location, it takes a positive value *α* for bright-bar detection and a negative value for dark-bar detection (any value ≠0 can be chosen because it merely rescales the input to the difference rule, leaving the output binary choice unaffected). *r* is therefore $\alpha (s_{7}^{\left[1\right]}-s_{7}^{\left[0\right]})$. Across trials, the average differential response to target minus non-target in the bright-bar detection task is *μ*(*r*) = *αρ*^[1]^*σ*_*N*_, and the standard deviation of the response to noise alone is *ασ*_*N*_. Ideal *d*′ (ratio of the two quantities just described) is therefore simply equal to *ρ*^[1]^, the SNR. Efficiency is defined as the square ratio between human and ideal *d*′ [29].

### Computational models

We selected a range of architectures to span commonly adopted models [6, 14, 30, 56], together with a novel variant (two-branch model) that accounts for our results. For each model, we present simulated results from selected parameterizations that most closely approximate our empirical descriptors, however model parameterization is not a critical factor in excluding specific models. We exclude models based on what we term ‘structural failures’ [14], i.e. failures to capture specific features of the data in binary fail/succeed fashion, rather than in relation to the size of those features. For example, if we measure a positive trend for contrast dependence of sharpness, a given model is excluded if unable to produce a similarly positive trend (any trend produced by the model must be either null or negative). For the models we consider, this type of structural failure is predictable on the basis of theoretical considerations about model architecture, regardless of parameterization. For example, the template matcher model is not expected to demonstrate any SNR/contrast dependence of linearity index (see above).

#### Linear branch

All models generate psychophysical decisions via the difference rule [32] detailed earlier, and apply front-end filter **f** to the stimulus. **f** is sampled at the same resolution used for the stimulus, i.e. at linearly arranged spatial positions *x*_*i*_ separated by 9 arcmin; it is defined by the difference-of-Gaussians (DOG) function *f*_*i*_ = *kϕ*(*x*_*i*_, *σ*_1_) − *ϕ*(*x*_*i*_, *σ*_2_) with SD values *σ*_1_ = 5 arcmin (positive component) and *σ*_2_ = 20 arcmin (negative component) for zero-mean Gaussian probability density function *ϕ*. Unless otherwise specified (see further below) *k* = 4. The choice of a DOG function is motivated by specific features of our data and by neurophysiological evidence [57], however it is not critical to our simulations provided its replacement displays the customary Mexican-hat shape [1]. **f** is normalized to unit-energy and applied via convolution (*) to generate the output of the *linear* branch **b**_lin_ = **s** * **f**. This application of **f** to the stimulus differs from the template-matching operation defined by the ideal observer (although both are linear operations), hence **b** is a vector, not a scalar. When gain-controlled, this output is ${\hat{b}}_{\text{lin}}=\frac{b_{\text{lin}}}{10+{\left(\sum b_{\text{lin}}\right)}^{2}}$ where ∑**x** sums values across vector **x**, and the hat notation ($\hat{b}$) is used to indicate that the relevant quantity is normalized via gain control.

#### Nonlinear branch

The output of the *nonlinear* branch is $b_{\text{nlin}}=b_{\text{lin}}^{2}$ where squaring is applied separately to each element of **b**_lin_. When gain-controlled ${\hat{b}}_{\text{nlin}}=\frac{b_{\text{nlin}}}{0.4+\sum b_{\text{nlin}}}$. The only substantial difference between the two branches is therefore dictated by the location at which squaring is applied along each branch. Along the linear branch, squaring is only applied within the gain-control component and only acts on the integrated output of the convolutional layer (i.e. it never acts on the output of individual filters within the layer). Along the nonlinear branch, squaring is applied immediately after convolution, acting on each value of the convolutional output. It is the introduction of this early nonlinear operation that confers highly nonlinear properties to the branch. Even when gain-controlled, the linear branch cannot display an equivalent degree of departure from linearity.

#### Read-out stage (including MAX)

The final output of the two-branch model to one stimulus is $r=\langle w,{\hat{b}}_{\text{lin}}+{\hat{b}}_{\text{nlin}}+e\rangle$ where the hat notation may be omitted to indicate that gain control is not applied, **w** is a Gaussian weighting function of SD *σ*_w_ = 40 arcmin akin to an attentional window [20], and each element of **e** is defined by a Gaussian noise source with a standard deviation that is chosen to yield d′ ∼1 (threshold) averaged across SNR and contrast conditions. It is important that the intensity of this internal noise source is *fixed* for a given model, i.e. it is *not* arbitrarily modified for different stimuli submitted to the same model. The same applies to all other model parameters except *σ*_w_ in the MAX model. For the MAX model, the decision variable is *r* = max(**w** × **b**_lin_ + **e**) and *σ*_w_ is set to 40, 80, 120 and 160 arcmin as contrast level decreases from high (narrow attentional window) to low (broad attentional window). The above-detailed change in *σ*_w_ for the MAX model is entirely arbitrary, i.e. it is not produced by the model itself as contrast is varied, but simply plugged into the model by the modeller.

#### Variants of two-branch model

We consider 5 variants of the two-branch model detailed above (in addition to the MAX model). In variant #1, only the linear branch (without normalization) contributes to the final response, i.e. *r* = 〈**w**, **b**_lin_ + **e**〉. This expression reduces to a noisy template matcher model, for which it is possible to formulate specific predictions (e.g. λ^[1]^ = λ^[0]^) that are in conflict with the empirical measurements [22–24]. A simplified neuronal analogy is the simple cell [58], although we know that actual simple cells display specific characteristics that are not accounted for by the notion of a linear template, in particular gain control [59]. In our simulations, the addition of gain control to the linear branch (so that the response is $r=\langle w,{\hat{b}}_{\text{lin}}+e\rangle$) does not rectify the failures associated with this branch in isolation. In variant #2, only the nonlinear branch (without normalization) contributes to the response, i.e. *r* = 〈**w**, **b**_nlin_ + **e**〉. A simplified neuronal analogy is the complex cell [58]. In variant #3, the nonlinear branch is gain-controlled so that $r=\langle w,{\hat{b}}_{\text{nlin}}+e\rangle$. This variant is particularly relevant to existing literature because it represents a more realistic implementation of an idealized complex cell [59], and because it is the most commonly adopted model in psychophysical accounts of the well-known dipper effect [60].

Variants #4-#5 combine linear and nonlinear branches. In variant #4, only the nonlinear branch is gain-controlled ($r=\langle w,b_{\text{lin}}+{\hat{b}}_{\text{nlin}}+e\rangle$). In variant #5, both linear and nonlinear branches are gain-controlled ($r=\langle w,{\hat{b}}_{\text{lin}}+{\hat{b}}_{\text{nlin}}+e\rangle$). Two-branch models of this kind are less common in the literature, however there are notable exceptions [39, 61, 62] (see Discussion). For variant #5, we used slightly different parameters (*k* = 6, *σ*_1_ = 2.5 arcmin, *σ*_2_ = 10 arcmin, *σ*_w_ = 10 arcmin) to improve qualitative match with the data.

#### Early versus late intrinsic noise

As detailed above, internal noise is generally applied in the form of a late additive source of stimulus-decoupled variability [26]. The term ‘additive’ is *not* used here in relation to the distinction between ‘additive’ and ‘multiplicative’ noise: when these two terms are adopted in the literature, multiplicative noise is still represented by an additive term; the difference is that, in the case of ‘additive’ noise, the intensity of the additive source does *not* scale with the overall responsivity of the mechanism to which it is added, whereas in the case of ‘multiplicative’ noise, the intensity of the additive source does scale with responsivity by a fixed factor [63] (related to the Fano factor [64]) or, in some formulations, via more articulated rules [65]. To avoid confusion, below we adopt the terms ‘response-independent’ and ‘response-dependent’ to substitute for ‘additive’ and ‘multiplicative’ (see [66] for a similar choice of terminology), and retain additive in the sense of merely adding the noise term onto the stimulus-driven response generated by the model.

In psychophysics, the distinction between ‘response-independent’ and ‘response-dependent’ noise is difficult to ascertain because there is no absolute scale for ‘responsivity’ along the decision variable (as explained in the section detailing internal noise estimation). For example, if it is found that internal noise intensity varies from 1 to 2 between conditions A and B, this may happen because the stimulus-driven responsivity inside the participant’s head has halved, or because the stimulus-decoupled internal variability has doubled. Similarly, if it is found that internal noise intensity remains unchanged between conditions A and B, this may nevertheless result from changes in responsivity and variability that scale with each other (e.g. responsivity doubles and so does variability). Notwithstanding these difficulties in interpreting behavioural measurements of internal noise, the depth of the empirical characterization adopted in this study supports some meaningful inferences regarding the trends predicted by different types of intrinsic variability (see below).

The response-independent-versus-dependent dichotomy is often studied alongside the distinction between ‘early’ and ‘late’ sources [67, 68]. We consider both factors in relation to the main sub-components of our modelling strategy, namely linear and nonlinear branches. With relation to the linear branch, these issues are immaterial: there is no distinction between early and late noise, because all additive terms can be grouped into a single additive term equivalent to the late noise source we have adopted in all our simulations. Similarly, the distinction between response-independent and response-dependent noise is of little interest because, for a given stimulus regime (e.g. fixed contrast and SNR), the nature of the noise source is equivalent (i.e. additive with same distribution, despite potentially having different scale). No tuning changes are theoretically expected for the linear branch [22, 23], regardless of whether noise is early/late or response-independent/dependent. We therefore focus our attention on the nonlinear branch.

Along the nonlinear branch, there are only two non-equivalent locations where noise may be added: before the squaring nonlinearity (early noise), or after it (late noise, already detailed above). Early noise is simulated by adding **e** to the output of the front-end convolutional layer *before* squaring: (**s** * **f** + **e**)^2^ (as opposed to the late formulation (**s** * **f**)^2^ + **e** adopted previously). Response-dependent noise is simulated by rescaling noise intensity using the absolute values returned by the convolutional layer: if *b*_*i*_ is one element of vector **b** = **s** * **f**, corresponding to the response generated by one filter in the layer, noise term |*b*_*i*_|*e*_*i*_ is added to it, where *e*_*i*_ is one element of noise vector **e**. As for all simulations in this study, the SD of the Gaussian noise source is chosen to yield d′ ∼1 across conditions (see above).

We do not consider the role of intrinsic noise for more complex architectures than the nonlinear branch used here, because it is not parsimonious to do so: once we take the step of combining two branches as we have done here, we demonstrate that the simplest form of internal noise (fixed late additive) is sufficient to account for our experimental observations. We do not exclude that the response-independent-versus-dependent distinction, for example, may play a critical role in shaping SNR/contrast-dependence for specific highly articulated models, indeed our simulations show that early noise produces different trends for SNR dependence of the nonlinear branch depending on whether it is response-dependent or response-independent (as expected, and contrary to our empirical measurements, response-dependent noise predicts strong positive SNR-dependence with no contrast-dependence, see S3D and S3E Fig). Our goal here is to identify the most parsimonious modelling circuit for our results; within the context of this strategy, the critical factor appears to be the transition from a single-component to a double-component architecture [39, 61], rather than the specific manner in which intrinsic variability is introduced into the model.

### Second-order diagonal under MAX model

In prior work [20] we have derived an expression of the following form for the diagonal of the second-order descriptor under the MAX model (defined above): *k*(**f**^2^ ⋆ **w**) where *k* > 0 and ⋆ is cross-correlation. Any plausible implementation of MAX involves values ≥0 for the weighting function **w** (this function specifies weights on the output of the front-end filtering layer before the maximum operation; negative values do not correspond to a sensible/plausible perceptual mechanism in this context). Every entry of the second-order diagonal must therefore be ≥0, presenting the opportunity for robust empirical verification (i.e. this prediction may be falsified by demonstrating that empirical estimates of second-order descriptors present significant negative modulations along their diagonal, see S2 Fig).

### Dipper simulations

The reference model is the two-branch model detailed above with gain control on the nonlinear branch only (variant #4), without any change of parameterization. Both target and non-target stimuli are noiseless versions of the bright-bar stimulus (i.e. simply consist of a central bright bar) and only differ by intensity Δ*ρ*: *ρ*^[1]^ = *ρ*^[0]^ + Δ*ρ*, where *ρ*^[0]^ is pedestal intensity. We sample *ρ*^[0]^ over two orders of magnitude in 50 logarithmic steps including *ρ*^[0]^ = 0 (detection), and vary Δ*ρ* in steps of less than 5% average pedestal intensity over a range sufficient to measure a full psychometric curve (from chance to 100% correct). We ran 100 simulations of 1000 trials each for every choice of *ρ*^[0]^ and Δ*ρ*, and determined the Δ*ρ* value associated with a performance level of 75% (threshold) for each *ρ*^[0]^ value. The average Δ*ρ* value across simulations defines the threshold-versus-pedestal psychometric function [60]. We also repeated this process for each branch of the model separately, to measure branch-specific psychometric functions.

### Statistical analysis

We adopt a combination of confidence intervals and *p* values to avoid the limitations associated with *p* values alone [69, 70]. There are no instances in this study where these two approaches are in conflict. When comparing two datasets or one dataset against 0, *p* values are returned by two-tailed Wilcoxon signed-rank tests. When determining trends (e.g. across 4 ordinal stimulus levels), *p* values are returned by Friedman tests. The experiments were designed so that the null hypothesis (no departure from 0 or lack of trend) is transparently and unambiguously defined as involving no difference between two or more measurements of the same variable under different manipulations. In general, the primary effects reported in this study are sufficiently large and robust to eliminate any concern as to their statistical reliability. To verify robustness/replicability, we adopt a confirmatory approach where we tackle the primary result from multiple directions with additional experiments involving different specifications of target signals, visual field location, mode of stimulus delivery and assigned task. We find remarkable consistency across these varying conditions, supporting robust conclusions about the structure of our dataset.

## Results

### Overview of experimental findings

**Fig 1:**
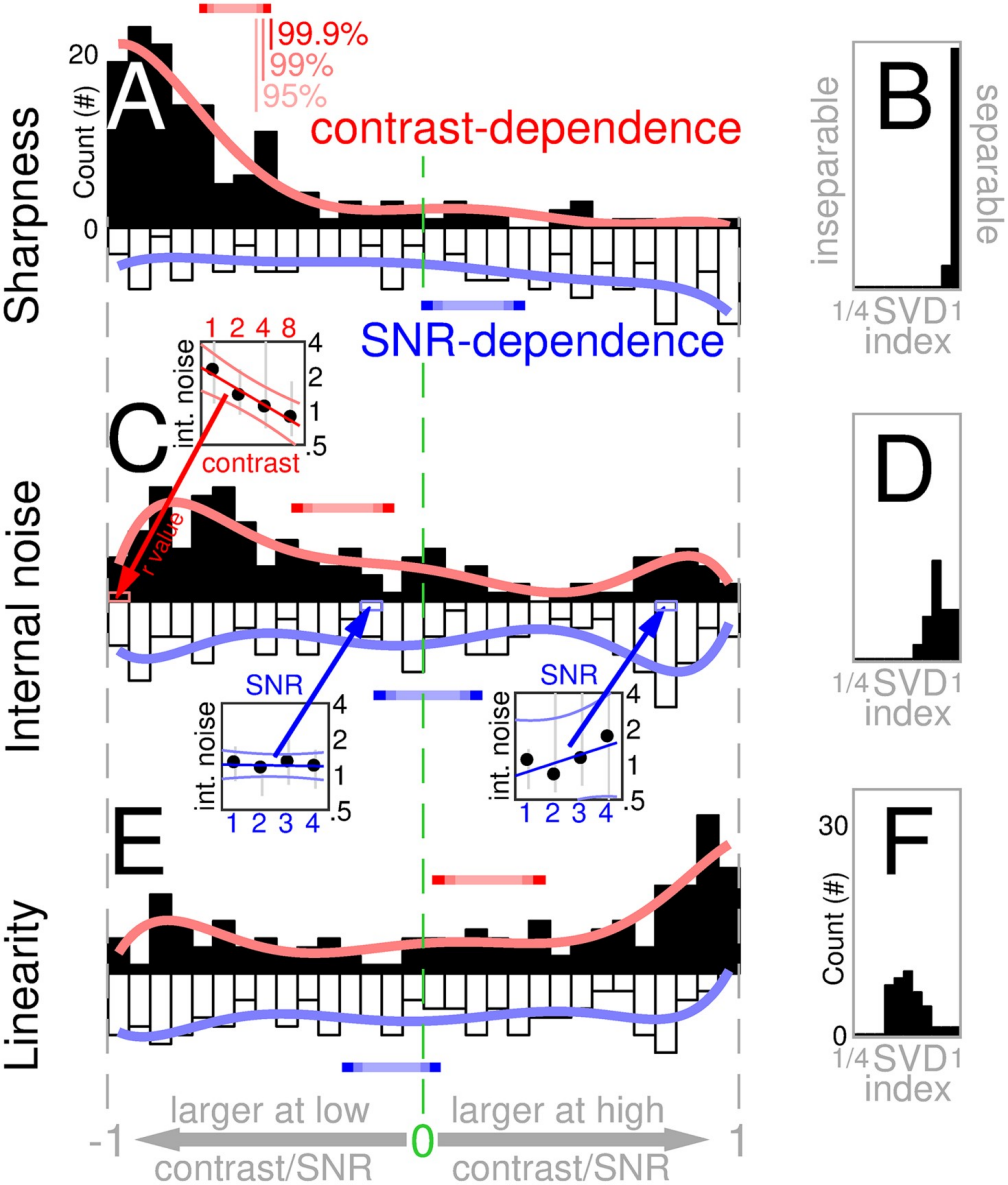
Increasing contrast reduces tuning sharpness, intrinsic noise and nonlinear processing. Each histogram in A,C,E plots the distribution of 148 independent estimates of correlation coefficients computed from entire dataset (>half a million trials); each estimate refers to a specific SNR/contrast level from a specific task in an individual observer. A plots distribution of contrast-dependence (solid bars, red labels) and SNR-dependence (open bars, blue labels) for sharpness of spatial tuning (smooth curves show polynomial fits of degree 6 for visualization purposes only); C,E plot same for internal noise and composite linearity index (see Methods for how these quantities were defined and measured). Ordinate plots number of estimates that fall within the corresponding bin on abscissa. Dependence (plotted on x axis) is the correlation coefficient between the manipulated variable (contrast or SNR) and the measured characteristic (sharpness, internal noise or linearity). Three square insets to panel C show three examples of how internal noise varied as a function of contrast (top inset with x axis labelled ‘contrast’) or SNR (bottom insets with x axis labelled ‘SNR’). Top inset plots internal noise values at four contrast levels (plotted on x axis) and SNR = 3 for one participant in the foveal bright-bar detection task; bottom-left inset plots values at four SNR levels (plotted on x axis) and contrast = 1% for one participant in the mixed-polarity detection task; bottom-right inset plots values at four SNR levels and contrast = 2% for one participant in the peripheral bright-bar detection task. In each inset, red/blue line shows linear fit, light-red/light-blue lines show associated 90% confidence intervals. From each inset, a correlation coefficient is computed and binned into the histograms in C as indicated by arrows. This process is repeated across the entire dataset, to produce the full distributions in A,C and E. When distribution is skewed to the left (e.g. solid bars in A), relevant characteristic (e.g. sharpness in A) is greater at low contrast (solid bars) or SNR (open bars); when distribution is skewed to the right (e.g. solid bars in E), relevant characteristic (e.g. linearity index in E) is greater at high contrast/SNR. Colour-graded red/blue horizontal bars mark confidence intervals for the mean (95/99/99.9% for low/mid/high colour saturation). B,D,F plot distributions from 37 independent estimates (one estimate from each observer and task) for separability of contrast- versus SNR-dependence (estimated using singular value decomposition (SVD); see Methods).

The main empirical results of this study are summarized in Fig 1, where data is pooled from all experimental conditions. We present this summary upfront to focus readers on the main features of interest that are common across conditions and that are critical to our main conclusions, with the understanding that this approach may overlook important differences between conditions. We then examine each experiment separately and highlight its distinguishing features and significance in relation to other experiments.

In this study we map perceptual filters for extracting a fully specified, well-localized, luminance-defined bar using established techniques [22, 23, 31]. The expected spatial profile of the relevant filter is known to be Mexican-hat-shaped from previous studies [71–73] and is primarily characterized by its ‘sharpness’, a quantity related to the presence of surround inhibition and bandpass tuning. We quantify sharpness using a robust validated metric (see Methods), and compute its correlation with two important stimulus parameters: contrast (modulation around background luminance) and signal-to-noise ratio (SNR: the ratio between the intensity of the bar to-be-detected and the superimposed noise modulation).

The resulting distributions of correlation coefficients are plotted in Fig 1A: top/solid/red when correlating against contrast, bottom/open/blue when correlating against SNR, which we refer to as ‘contrast-dependence’ and ‘SNR-dependence’ respectively (dependence therefore ranges between -1 and 1 on the x axis, being defined by a correlation coefficient; see Methods). There is a substantial (average coefficient -0.6) highly significant (p<10^−20^) shift of the distribution towards negative values for contrast-dependence (bottom histogram), together with a mild (∼0.16) less significant (p<0.01) shift towards positive values for SNR-dependence (top histogram). Confidence intervals (indicated by horizontal bars) show no overlap with 0 (dashed vertical green line) for contrast-dependence, while there is overlap at 99.9% confidence for SNR-dependence. A confidence interval of 99.9% may seem overly conservative, however the results in Fig 1 come from a very large data mass (>0.6 million trials), justifying a high threshold for evaluating any associated shift. The effects of contrast and SNR can be studied separately, as afforded by Fig 1A, because they act independently on sharpness, as we verified via a commonly adopted separability index (Fig 1B; see Methods).

We also measured internal noise: the variability in psychophysical response that is *not* coupled with the stimulus (i.e. intrinsic to the observer [74]). This quantity, like sharpness, is clearly contrast-dependent: although the associated correlation coefficient is on average smaller (-0.26), it remains statistically significant (p<10^−6^) at the most stringent confidence level (see red horizontal bar in Fig 1C). There is no overall dependence on SNR (p = 0.83; see also blue horizontal confidence bar).

Finally in relation to Fig 1, we estimate the degree of linearity demonstrated by the perceptual filter, where ‘linear’ is understood in connection with the notion of ‘template-matching’ [14, 38]. We find a marked (0.2) significant (p<10^−3^) positive contrast-dependence of this quantity, with no SNR-dependence (see Fig 1E, in particular red/blue confidence error bars). The effects of contrast and SNR on linearity demonstrate less separability than their effects on sharpness and internal noise (Fig 1F).

To summarize the above observations, stimulus contrast has a sizeable impact on all three metrics in Fig 1 (all red horizontal confidence bars stand clear of the green vertical dashed line marking no effect), while SNR produces smaller effects of dubious significance, sometimes none at all (Fig 1C; all blue horizontal confidence bars overlap with green vertical dashed line). The effect of increasing contrast is a *decrease* in sharpness and internal noise, together with *linearization* of system transduction. This summary account inevitably overlooks important features of our diverse dataset that cannot be subsumed under a common distribution (e.g. non-negligible inseparability in Fig 1F, potential bimodality of distributions in Fig 1C), prompting closer evaluation of each condition separately (see below).

### Human efficiency in task performance

**Fig 2:**
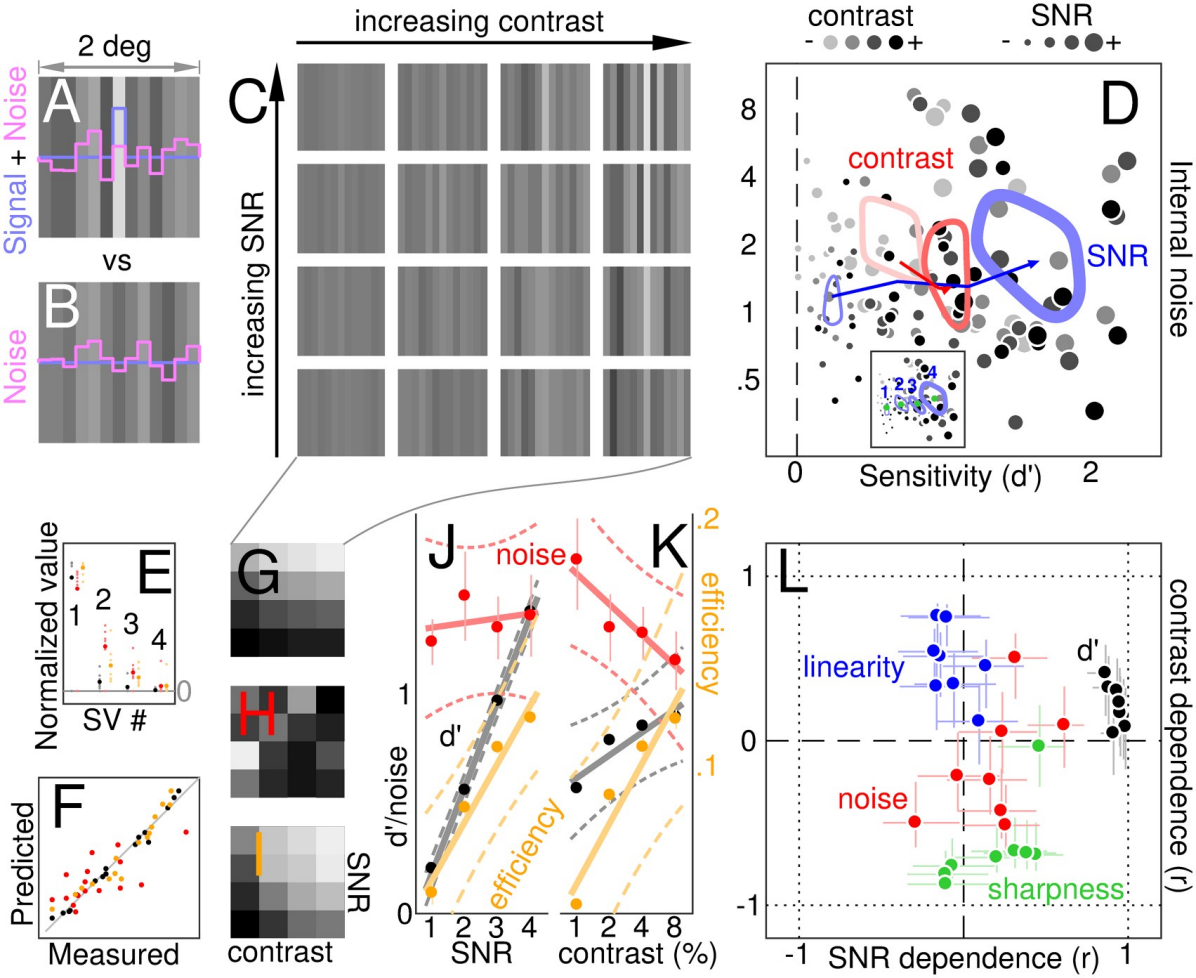
From visual stimulus to summary data descriptors. Observers were asked to discriminate between a signal+noise stimulus (A), containing a central bright bar (blue profile) superimposed onto luminance noise (magenta profile), and a noise-only stimulus (B). Contrast was manipulated by rescaling all luminance values (referenced to background luminance); signal-to-noise ratio (SNR) was manipulated by varying the ratio between luminance of the target signal (central bar) and standard deviation of the noise (see signal+noise example stimuli in C). Each dot in D plots internal noise (y axis) versus sensitivity (x axis) for each subject and each combination of contrast/SNR; dot size reflects SNR, dot colour reflects contrast (light→dark for increasing contrast). Contours demonstrate data spread at low/high contrast (light-red/dark-red) and low/high SNR (thin/thick blue lines); arrow stems join different mean levels from low to high as illustrated by the inset (digits refer to SNR levels 1-4 for the 4 different contours; green dots mark corresponding cluster mean values for each SNR, averaged across contrast levels). Each contour runs along the surface generated by blurring data points with a Gaussian point-spread function of SD equal to 20% of panel width, at a height equal to 80% of surface peak. G-I show sensitivity, internal noise and absolute efficiency for each of the 16 contrast/SNR combinations (mean across observers) as surface plots (increasing value from dark to bright). E plots singular values (SV) for surfaces in G-I (colour-coded as black for sensitivity, red for internal noise and orange for efficiency), demonstrating good degree of separability (large SV#1). F plots each value on each surface in G-I (x axis) against its prediction under separability (y axis). J-K plot marginal averages across the surfaces in G-I (solid lines show best linear fits, dashed lines show 95% confidence intervals around fits; black/red data are plotted to black numerical ordinate values, orange to orange values). L plots contrast dependence (y axis) versus SNR dependence (x axis) for sensitivity (black), internal noise (red), tuning sharpness (green) and linearity index (blue) separately for each participant (one dot per participant; dependence is defined by a correlationn coefficient (r), see insets to Fig 1C and its caption for examples of how it is calculated). Please refer to Methods for how these quantities were computed. Error bars in E,J-L plot ±1 SEM. Error bars are omitted in D to avoid clutter.

In the simplest variant of the experiment, observers reported the location (left versus right) of a signal+noise stimulus (Fig 2A) flashed to one side of fixation, as opposed to a noise-only stimulus (Fig 2B) simultaneously presented on the opposite side. Each stimulus consisted of 13 independently modulated luminance bars; signal profiles, alongside example noise profiles, are shown by blue/magenta lines in Fig 2A and 2B. Signal location was explicitly indicated by visible markers to minimize spatial uncertainty (see Methods). We varied both SNR and contrast across four levels for each characteristic (resulting in 16 possible SNR-contrast combinations illustrated in Fig 2C), with the specific SNR/contrast values chosen to equate efficiency across the range spanned by the two stimulus parameters (orange data in Fig 2J closely matches orange data in Fig 2K).

As expected [29], sensitivity (d′) scales linearly with SNR (black data in Fig 2J). The scatter plot in Fig 2D attempts to capture this effect without restricting access to individual-observer data: each data point refers to one observer responding to one of the 16 possible stimulus configurations. Symbol size (proportional to SNR) scales with sensitivity, as summarized by the blue arrow (pointing from low to high SNR). Increased contrast is also associated with enhanced sensitivity (red arrow/contours in Fig 2D and black data in Fig 2K), however this effect is substantially smaller: there is a ∼50% increase in sensitivity for the 8-fold increase in contrast, compared with a 7-fold increase for the 4-fold increase in SNR. The sensitivity range spanned by the contrast manipulation is ∼0.6-0.9, fully contained within the range spanned by the SNR manipulation (0.2-1.4). This apparently trivial result is critical to our study, because it enables us to exclude that contrast-dependent effects may be merely byproducts of concomitant variations in sensitivity (a serious confound for other studies e.g. [75]): should that be the case, we expect even larger effects for varying SNR, contrary to what we observe (see Fig 1; see also Methods).

The univariate plots in Fig 2J and 2K offer a succinct view of how sensitivity (black data) varies as a function of SNR and contrast separately, but it does not support inspection of potential interdependencies between these two characteristics. For example, it is possible that sensitivity scales with SNR at high contrast, but not at low contrast; this data structure would not be available from Fig 2J because, in that plot, sensitivity is averaged across contrast for each SNR value on the x axis. The most accessible format for gauging potential interdependencies is to plot sensitivity as surface intensity on a 4×4 grid corresponding to the 4×4 possible contrast×SNR stimulus combinations, as shown in Fig 2G. This surface is very much consistent with the associated prediction from independently combining the effect of contrast and SNR, as demonstrated by the excellent match between measured and predicted values in Fig 2F (black data points). The separability of the surface into independent SNR and contrast effects is confirmed by the presence of a disproportionately large first component from singular value decomposition (Fig 2E; see Methods).

In an effort to strike a compromise between access to individual-observer characteristics (Fig 2D) and readable visualization of data structure (Fig 2J and 2K), we compute dependence on both contrast and SNR via correlation coefficients (already adopted in Fig 1) and plot them on y and x axes in Fig 2L separately for different observers. Sensitivity correlates positively with both SNR and contrast (black data points scatter to the right of vertical dashed line and above horizontal dashed line), however the latter effect is smaller (consistent with the trends demonstrated by the black data points in Fig 2J and 2K). Fig 2L therefore provides a compact summary of relevant results without sacrificing access to data structure at the individual-observer level. We alert readers to the fact that all quantities in Fig 2L range between -1 and 1 because they do not represent actual values of the indicated characteristic (e.g. sensitivity), but the dependence of this characteristic on contrast or SNR in the form of a correlation coefficient (i.e. they represent the same quantity plotted on the x axis in Fig 1).

### Contrast dependence of internal noise

The theoretical underpinning of sensitivity as assessed by the d′ metric is intimately connected with the notion of internal noise [29]; indeed, our method for estimating internal noise relies on the signal detection theory (SDT) framework that supports the definition of d′ [25, 26]. For this reason, we plot the two metrics against each other in Fig 2D, and apply to internal noise the same visualization/analysis adopted for sensitivity. Data points in Fig 2D become increasingly dark (higher contrast) as one moves downward along the y axis (decreasing internal noise); this effect is primarily visible in the low sensitivity range, and corresponds to the downward direction of the red arrow. Although statistically significant (p<0.02 from Friedman test), its size may at first appear underwhelming: an 8-fold reduction in contrast produces a 40% increase of internal noise (red data in Fig 2K). This result, however, must be interpreted in relation to the documented stubborness of this metric against displaying any systematic dependence on other important perceptual attributes [26] such as uncertainty [20], learning [76], and most relevant to the present study SNR [20], as we document here (red data in Fig 2J). We are not aware of any previous study reporting a systematic dependence of behavioural consistency on specific stimulus characteristics.

There is a sense in which the lack of SNR dependence is more surprising than the presence of contrast dependence. Internal noise is estimated via reverse application of SDT to two empirically measured quantities [25, 26]: the percentage of correct responses (% correct), and the percentage of matching responses to repeated presentation of identical stimuli (% agreement; see Methods). With increasing SNR, % correct naturally increases and does so substantially; in order for the resulting estimate of internal noise to remain constant, % agreement must also increase, and must do so specifically by the amount predicted by an SDT model with constant internal noise [25]. This means that, in order for the red data in Fig 2J to remain constant, % agreement must track % correct in line with the SDT prediction across a 4-fold change in SNR, which is not at all a trivial result. If any artefactual change in internal noise is to be expected from technical glitches of our estimation procedure, it would be expected under SNR manipulation and not contrast variations, because the latter cause smaller changes in % correct. The observed lack of SNR dependence combines with the measured contrast dependence to emphasize both the robustness of our estimation procedure and the surprising nature of the contrast-driven effects.

### Contrast dependence of tuning sharpness

**Fig 3:**
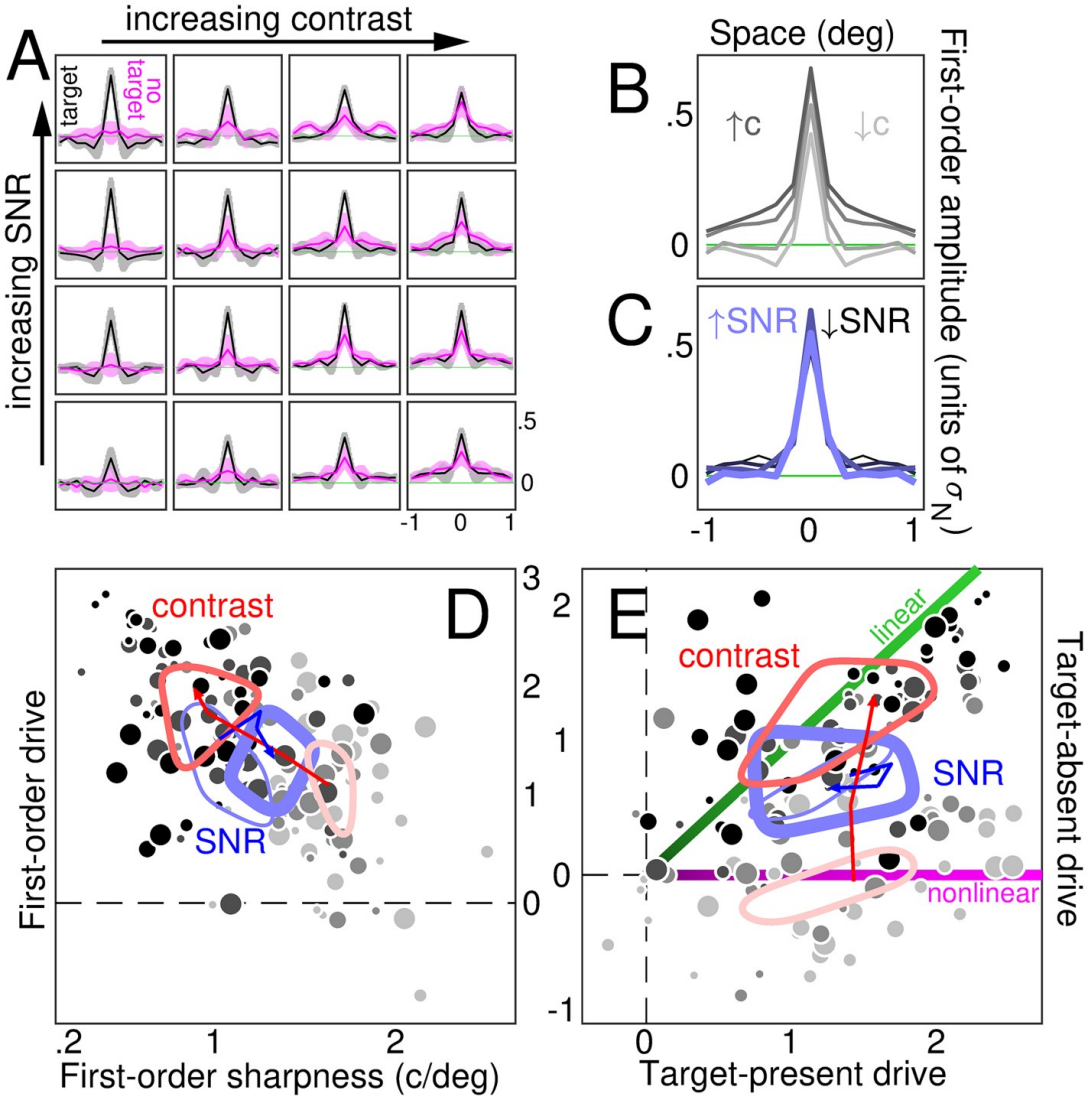
Contrast-driven retuning and linearization. A plots first-order tuning descriptors derived from noise samples with/without added target signal (black/magenta) for increasing contrast/SNR (x/y axes). Shaded regions show ±1 SEM. B shows full first-order descriptor for increasing contrast levels (grey→black), C shows same for increasing SNR (black→blue). D plots first-order drive (y axis) versus sharpness (x axis) to the plotting conventions of Fig 2D. E plots first-order drive for target-absent (y axis) versus target-present (x axis) descriptors to the same plotting conventions.

The two metrics of sensitivity and internal noise are essential descriptors of perceptual processing [74], however they provide an incomplete picture of the underlying mechanisms: judging from Fig 2J and 2K, one may conclude that the only qualitative difference between manipulating contrast and SNR is that the former, but not the latter, is associated with changes in internal noise. As we document below, there are many more qualitative differences between these two manipulations. To expose these differences, we rely on experimental descriptors that capture the tuning characteristics of the perceptual process [22, 23]: Fig 3B and 3C show spatial weighting profiles for extracting the central bar signal and their dependence on contrast (**B**) as well as SNR (**C**). It is apparent that SNR manipulations produce no appreciable change in tuning characteristics, while varying contrast has a profound impact on the shape of the spatial filter: as contrast varies from high (darker traces in Fig 3B) to low (lighter traces), the tuning function becomes sharper and lateral inhibition (completely absent at high contrast) confers the typical Mexican-hat shape commonly observed in sensory processing [1, 3].

We extract two metrics from each spatial tuning descriptor: its sensory-driven content normalized against background noise (see Methods) which we refer to as *drive*, reflecting the amount of structured modulation (if only noise, this metric equals 0); the centroid of its power spectrum across spatial frequency, reflecting its tuning *sharpness* (if lowpass, this metric is small, if bandpass/highpass, this metric is large; see Methods). When plotted against each other (Fig 3D) these two quantities are mildly correlated (r = -0.49, p<10^8^); neither depends on stimulus SNR (p>0.05, symbol size shows no obvious trend) but both depend on contrast (p<10^4^, see light/dark gradient of data points). Of particular interest is the contrast-dependence of sharpness (p<10^8^), demonstrated by the dark/light segregation of data points across the x axis, corresponding to greater sharpness (larger x values) at low contrast (lighter symbols), and further emphasized by the cluster separation indicated by dark-red and light-red contours (with associated red arrow). The contrast-dependence of sharpness, alongside the lack of SNR-dependence, is compactly summarized by the green symbols in Fig 2L using the conventions previously adopted for sensitivity and internal noise.

We emphasize that, although our stimuli were largely above visibility threshold, the above masurements pertain to discriminability of threshold signals, thus urging caution in attempting links with existing measurements of perceived contrast matching in the suprathreshold regime. It is well known that, above visibility threshold, low-contrast patterns appear finer (i.e. higher spatial frequency) than matched stimuli of higher contrast [77]. This perceptual effect (and related phenomena [78]) is classically interpreted/modelled via the notion of ‘labelled lines’ [17], combined with assumed *positive* contrast-dependence of spatial resolution for the underlying filters [77]. In the labelled line theory, a stimulus grating of 6 cycles/deg spatial frequency is perceived to modulate at 6 cycles/deg because it activates most strongly the filter associated with the 6 cycles/deg perceptual label; at high contrast, it is assumed that said filter is centred on 6 cycles/deg. At low contrast, however, it is assumed that the filter shifts its preference towards a lower frequency, say 4 cycles/deg. When a low-contrast grating of 4 cycles/deg spatial frequency is presented, it will activate the 6 cycles/deg perceptual label (now associated with a filter of lower preferred spatial frequency), leading to a percept of higher spatial frequency (6 cycles/deg instead of 4 cycles/deg). Our measurements of tuning characteristics go in the opposite direction (shift towards higher spatial frequency with decreasing contrast), but this inconsistency may simply reflect a fundamental difference between threshold discrimination and suprathreshold perception. If instead the two phenomena are connected, our results call into question some of the assumptions normally adopted to characterize suprathreshold perception.

Whenever a multivariate empirical descriptor (such as the spatial filters measured here) is reduced to a scalar quantity, the resulting surrogate descriptor (sharpness) is potentially subject to interpretational pitfalls. For example, it is possible to conceive tuning shapes that may look different in relation to what we may qualitatively term ‘sharpness’, and yet produce similar values of sharpness as defined by our surrogate metric. Other scalar descriptors may obviate the pitfalls associated with our chosen metric, but will introduce other potential pitfalls of a different nature. For this reason, correct interpretation of sharpness estimates must be accompanied by visual inspection of full tuning descriptors. It is evident from visual inspection of Fig 3B and 3C that the trend captured by sharpness estimates relates to the appearance of negative flanks at low contrast.

### Contrast-driven transition from nonlinear to linear regimes

The default framework for interpreting sensory transduction is template matching [23, 79] (see Methods for definition). Any departure from this model signals the presence of nonlinear processes that are not captured by the linear template, as has often been observed in previous studies [41, 45, 80, 81]. We assess potential departures using two data-driven metrics (see Methods for detailed explanation of how they are computed). The first metric involves a direct comparison between tuning descriptors derived from noise samples associated with the target signal (‘target-present’) and those derived from noise samples that did not contain the target (‘target-absent’) [14, 45, 81]. If the perceptual system behaves like a template matcher, we expect these two estimates to be the same and data points to scatter along the green diagonal equality line in Fig 3E; if the system departs from the template-matching prediction, we expect data points to scatter along the magenta horizontal line. It is evident that these two operating modes are associated with the transition from high to low contrast (dark-red versus light-red contours in Fig 3E). This transition is easily confirmed by statistical test (p< 10^−11^).

**Fig 4:**
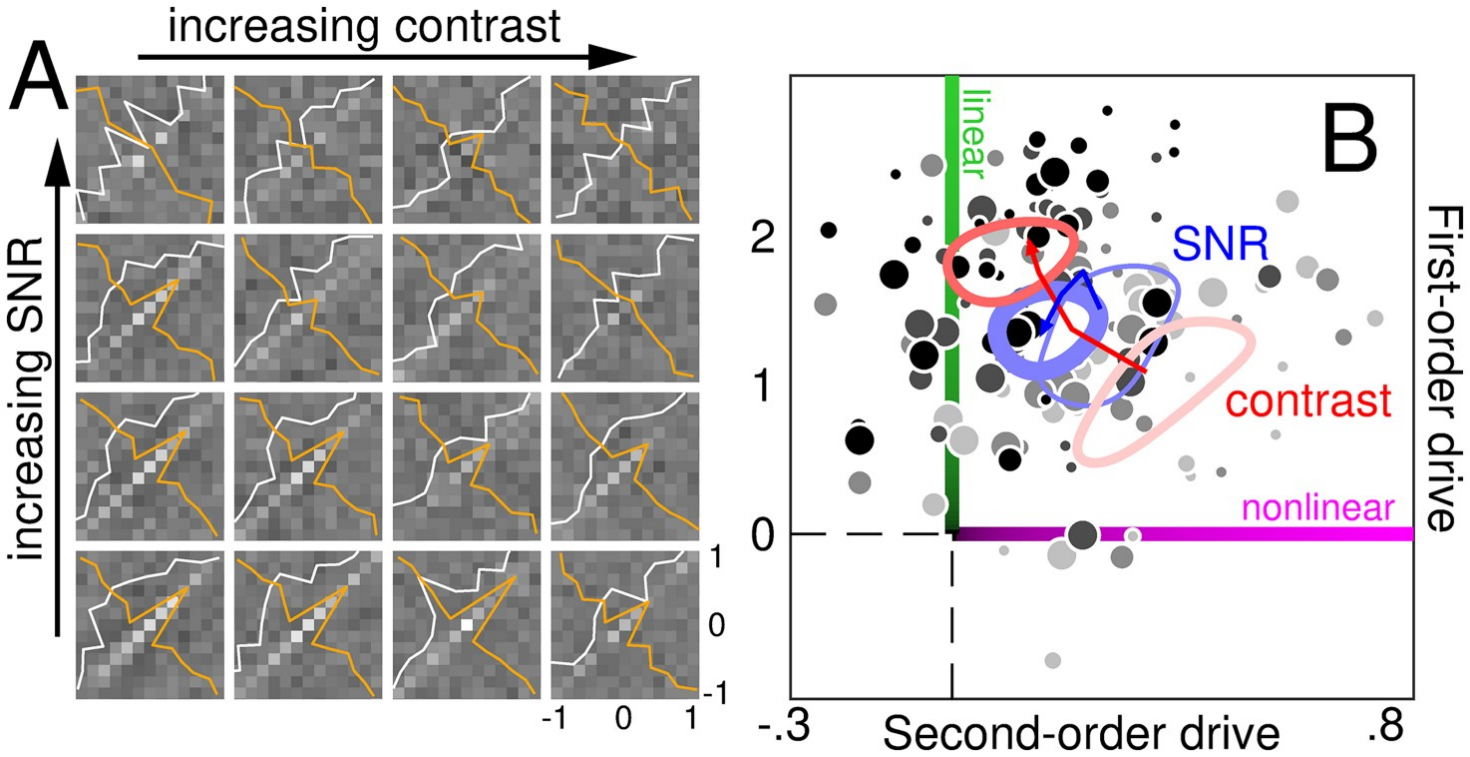
Second-order indicators of contrast-driven linearization. A plots second-order tuning descriptors for increasing contrast/SNR (x/y axes). White/orange traces show marginal averages along negative/positive diagonal directions. B plots first-order drive (y axis) versus second-order drive (x axis) to the plotting conventions of Fig 2D.

The second metric involves computing descriptors from the second-order statistical properties (i.e. covariance) of the noise samples classified by human observers (see Methods). The resulting estimates are shown in Fig 4A: it is evident that they modulate more at low contrast (left panels) than high contrast (right panels). Fig 4B plots drive from second-/first-order descriptors on x/y axes to separate linear and nonlinear regimes on a scatter characteristic analogous to Fig 3E, exposing a similar transition with increasing contrast (red arrow). Because this analysis is entirely consistent with the target-present/target-absent analysis above, we combine them into a composite linearity index (see Methods) and plot its contrast/SNR dependence compactly in Fig 2L (blue data points), demonstrating marked contrast dependence (p<10^−7^) with no SNR-dependence (p>0.2).

The nonlinear→linear transition raises an important issue, namely how exactly our spatial tuning estimates translate into tuning across spatial frequency. This issue is relevant to the manner in which the transduction characteristics of sensory devices are commonly characterized, i.e. in terms of frequency tuning [3, 4]. Because our sharpness index is based on spectral centroid analysis, it may be argued that we have already established a connection with the frequency-based description, however this is not entirely correct for two reasons. First, we demonstrate that system processing is qualitatively different between low and high contrast (see immediately above), rendering the connection between spatial filter and frequency tuning too opaque to assess using intuition alone. Second, we derive spectral centroids by computing power *not* from individual noise samples, but from their classified average. This procedure may overlook certain frequency-selective transduction properties, in particular those that are not phase-locked and would average to zero across space [45, 82].

**Fig 5:**
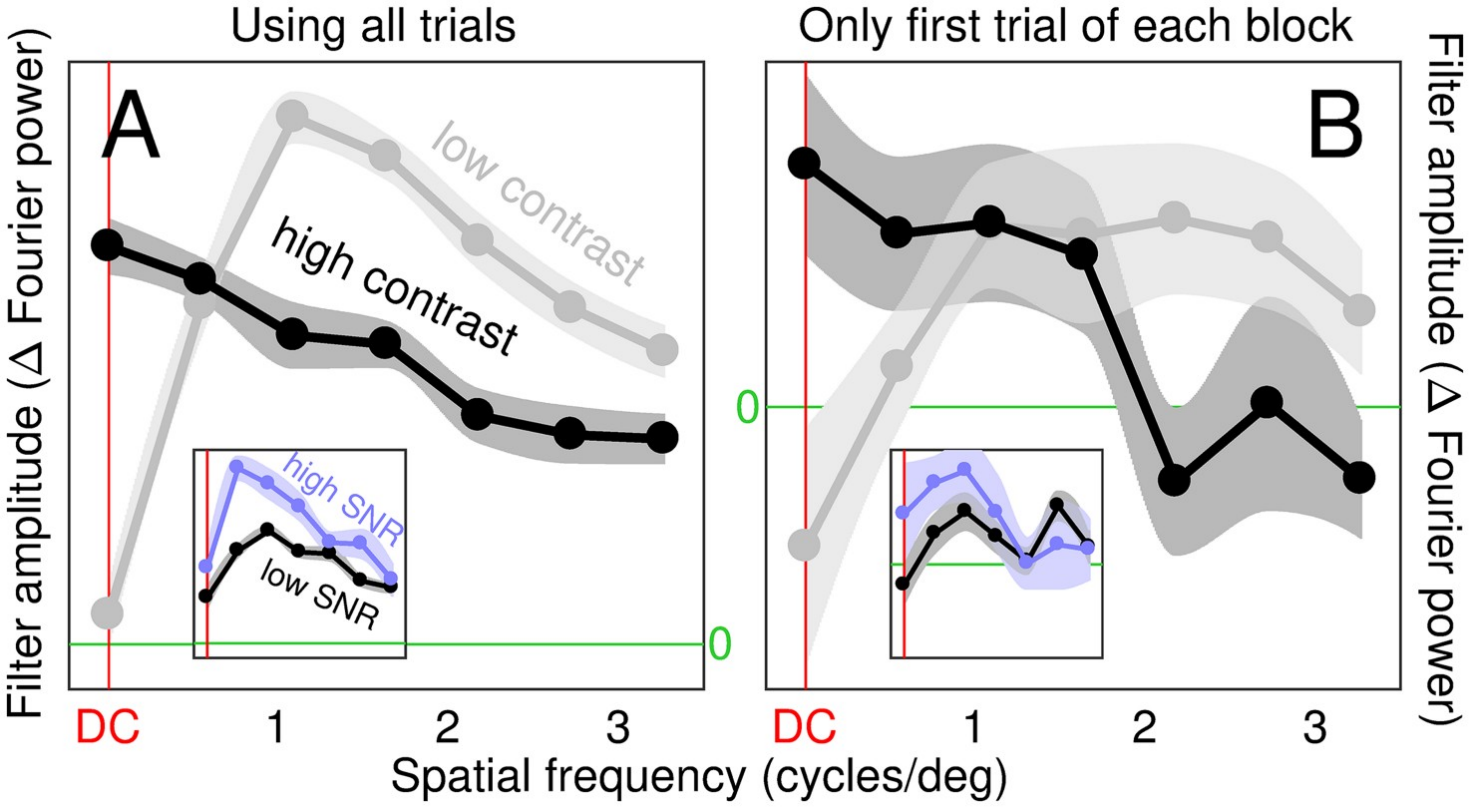
Contrast-driven retuning in Fourier power space. Tuning descriptors were computed with respect to Fourier power (y axis, arbitrary units) of individual noise samples across spatial frequency (x axis; see Methods). A plots traces for highest (dark) and lowest (gray) contrast levels; inset plots traces for highest (blue) and lowest (black) SNR levels. B plots same but only from first trial of each block (i.e. in the absence of memory, expectation and/or sequential effects).

To address the above issue explicitly, we recompute tuning estimates from noise samples individually converted to power spectra. As demonstrated in Fig 5A, the bandpass-to-lowpass transition with increasing contrast is just as evident. The notion that tuning becomes sharper at lower contrast is therefore *not* the specious result of unconvential data presentation/analysis: it remains valid when recast using established frameworks such as the frequency transfer function [3]. We also note that the spatial frequency range spanned by the data in Fig 5 closely matches expected values from relevant cortical measurements [83].

We also address here the issue of whether the bandpass→lowpass transition depends on observers’ expectations about upcoming stimulus characteristics: on each block, any given contrast/SNR stimulus specification was repeated across trials (see Methods); after the first trial, observers could gauge (at least approximately) the chosen SNR/contrast levels and potentially adjust their sensory circuitry to the expected stimulus characteristics. To evaluate the applicability (or lack thereof) of this scenario, we restrict our analysis to the first trial of each block; despite the drastic reduction in data mass (2%), we are still able to resolve the bandpass→lowpass transition (Fig 5B), demonstrating that it does not depend on mode of stimulus delivery and, more importantly, not on any potentially associated attentional/cognitive state (including trial-by-trial sequential dependencies [84]): the effects we observe reflect low-level sensory processing, *not* higher-level factors.

### Task variant designed to enforce energy computation

**Fig 6:**
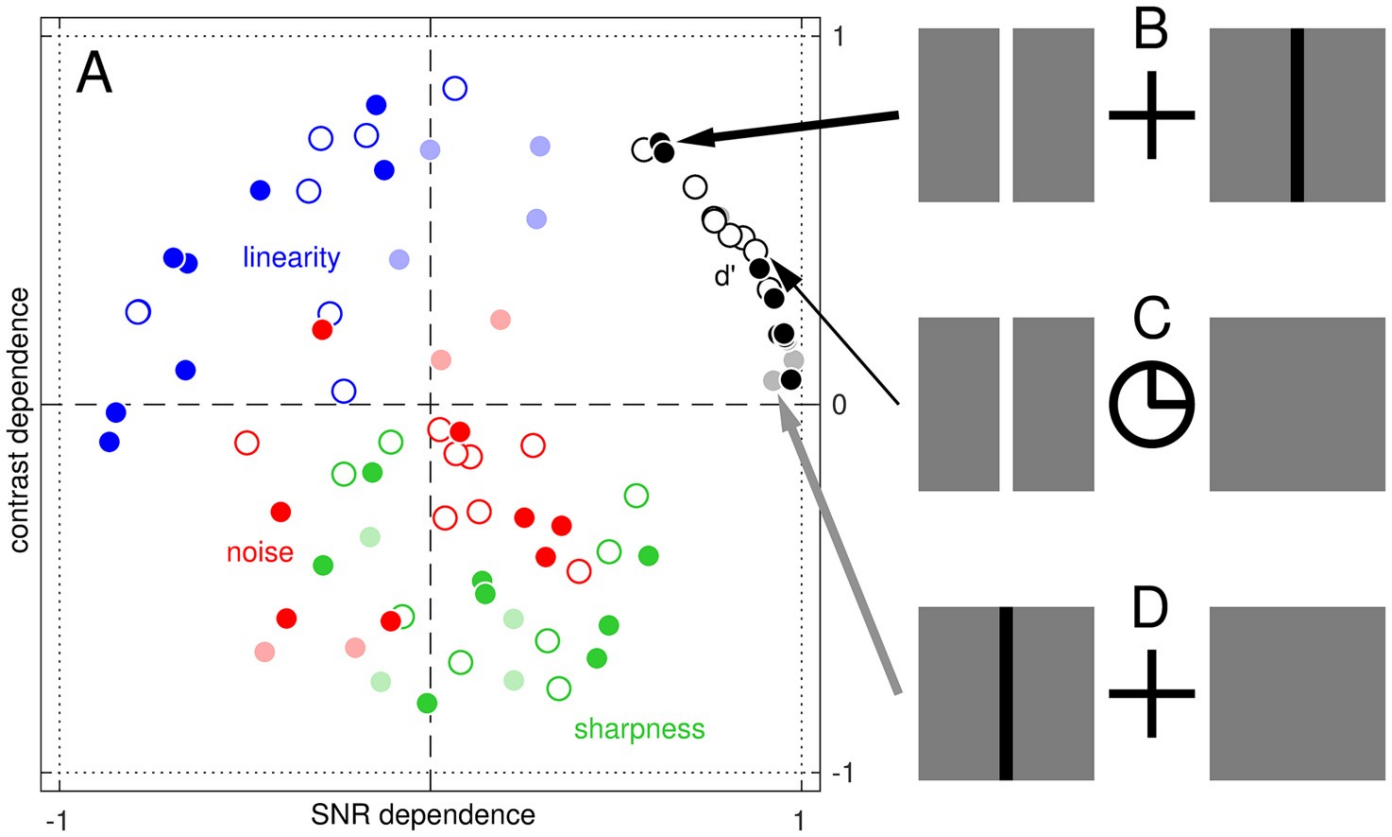
Main effects survive across a range of stimulus/task specifications. A is plotted to the same conventions of Fig 2L. Error bars are omitted to avoid clutter. Solid dark-color symbols refer to the polarity-discrimination task between stimulus icon on the left (target) and stimulus icon on the right (non-target) in B, simultaneously presented to opposite sides of fixation (central cross). Open symbols refer to the foveal variant of the bright-bar detection task in C for which both target and non-target (left/right icons) were displayed at fixation, but in temporal succession (clock icon in place of fixation cross). Solid light-color symbols refer to the dark-bar detection task in D.

The type of nonlinear processing exposed in Figs 2L, 3 and 4 is consistent with an operation akin to energy extraction as may be implemented by a complex-cell layer [24, 37]. Human observers switch between this operation (at low contrast) and a quasi-linear operation akin to template-matching (at higher contrast). The signal-to-be-detected in Fig 2A and 2B is visible to both operations, so that in principle observers may choose to rely on either one and still perform above chance. As we demonstrate with additional experiments, the same results are obtained when the target bar is dark rather than bright (light-coloured data points in Fig 6A) except for the expected sign-inversion of first-order descriptors [20] (S4 Fig).

An important question is whether observers would still engage both linear (template-matching) and nonlinear (energy-extraction) operations when faced with a task that can only be peformed by one of the two strategies. To answer this question with data, we designed a mixed-polarity variant of the experiment where the signal-to-be-detected is invisible to template-matching: the noise-only stimulus remains unchanged, but the signal+noise stimulus contains a signal that can either be bright *or* dark on any given trial, with bright/dark polarity assigned randomly from trial to trial (see Methods). Because the average signal across trials is 0, this signal cannot be detected by a linear mechanism.

**Fig 7:**
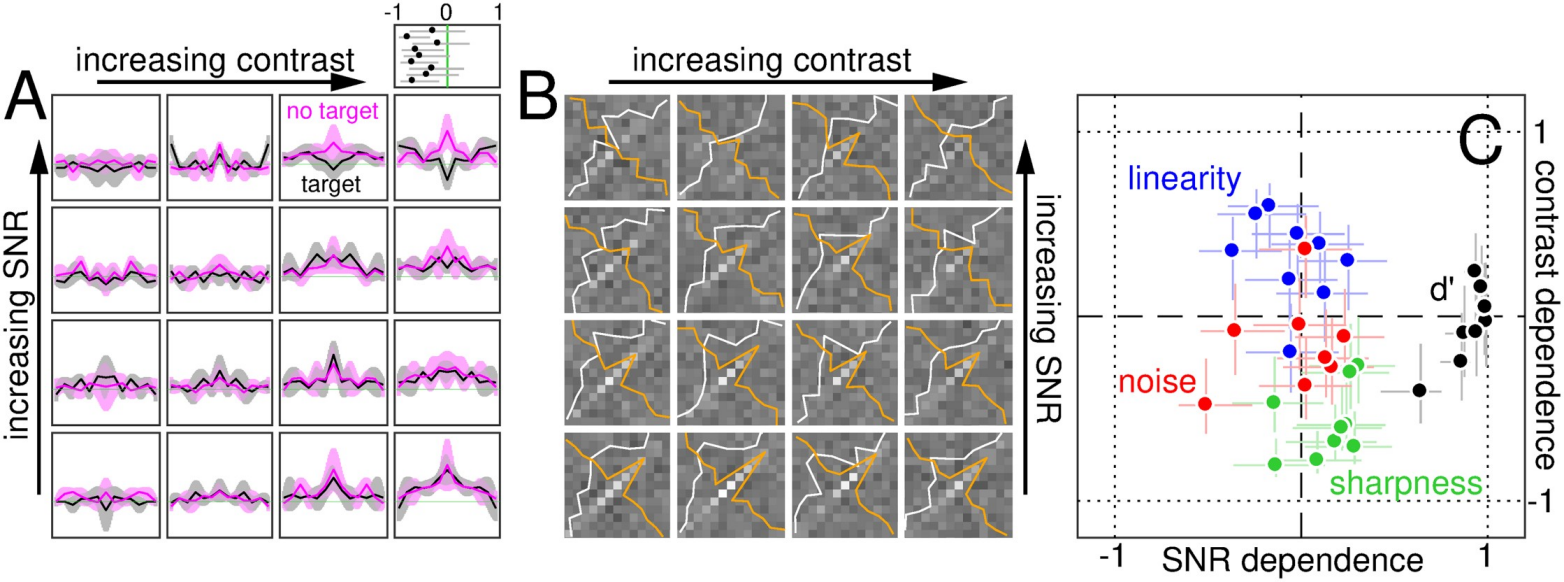
Idiosyncratic features of energy-extraction task. This dataset refers to discrimination between a target stimulus containing a bright *or* dark bar (mixed polarity), versus a non-target blank stimulus. A is plotted to the conventions of Fig 3A; B to the conventions of Fig 4A; C to the conventions of Fig 2L. As summarized in C, most trends observed with other tasks (Fig 6) are preserved, except d′ does not show contrast-dependence any longer (black symbols in C scatter around horizontal dashed line) and target-present/target-absent descriptors present an unusual inversion along the y axis (black/magenta traces at top-right in A; dots within plot immediately above show correlation between black and magenta traces for individual observers, where negative correlation (plotted on x axis) corresponds to mirror-inversion).

The main effects stand (Fig 7C). In particular, internal noise becomes larger at low contrast (p<0.02), sharpness becomes more pronounced (p< 10^−10^) and system processing switches to nonlinear (p< 10^−7^). However, this dataset presents interesting pecularities that set it apart from its counterpart with fixed-polarity target. As expected, first-order descriptors are greatly reduced in amplitude to the extent that they are virtually featureless at low contrast (left-most panels in Fig 7A). Another peculiarity is the puzzling amplitude inversion of target-present/absent profiles at high SNR/contrast (compare black/magenta traces within top-right subpanel in Fig 7A). The anticorrelated nature of the two descriptors is robust across observers (see plot above top-right subpanel where point-by-point correlation coefficients are shifted towards negative values at p<0.01 across participants) and is wholly counter-intuitive. We can account for it via computational modelling (see below) but we are unable to provide a transparent interpretation for this effect.

Finally, it is interesting that sensitivity for the mixed-polarity task does not improve with contrast (black data points scatter around horizontal dashed line in Fig 7C at p = 0.65). This result is unexpected in that the task involves minimal alterations of stimulus design from the conditions adopted in the other experiments, where we invariably found contrast-dependence (e.g. black symbols in Figs 2L and 6A). Thanks to the similarity in stimulus/task design, this result poses an interesting challenge for (and imposes an important constraint on) viable computational models, as they must be able to account for both presence and absence of contrast-dependence within a unified framework.

### Task variant designed to enforce template matching

The complementary task/stimulus design to the mixed-target protocol is one where the target stimulus contains a bright signal, while the non-target stimulus contains a dark signal of equal amplitude. This design is identical to the fixed-polarity protocol except the noise-only stimulus does not contain merely noise but also a non-target signal which, when processed by an energy extractor, cannot be discriminated from the target signal. The observer must engage a polarity-sensitive mechanism, such as template-matching, in order to perform above chance. The results of this additional experiment (solid dark-colour data points in Fig 6A) are very similar to those obtained in the main experiment (Fig 2L; see also S5 Fig).

**Fig 8:**
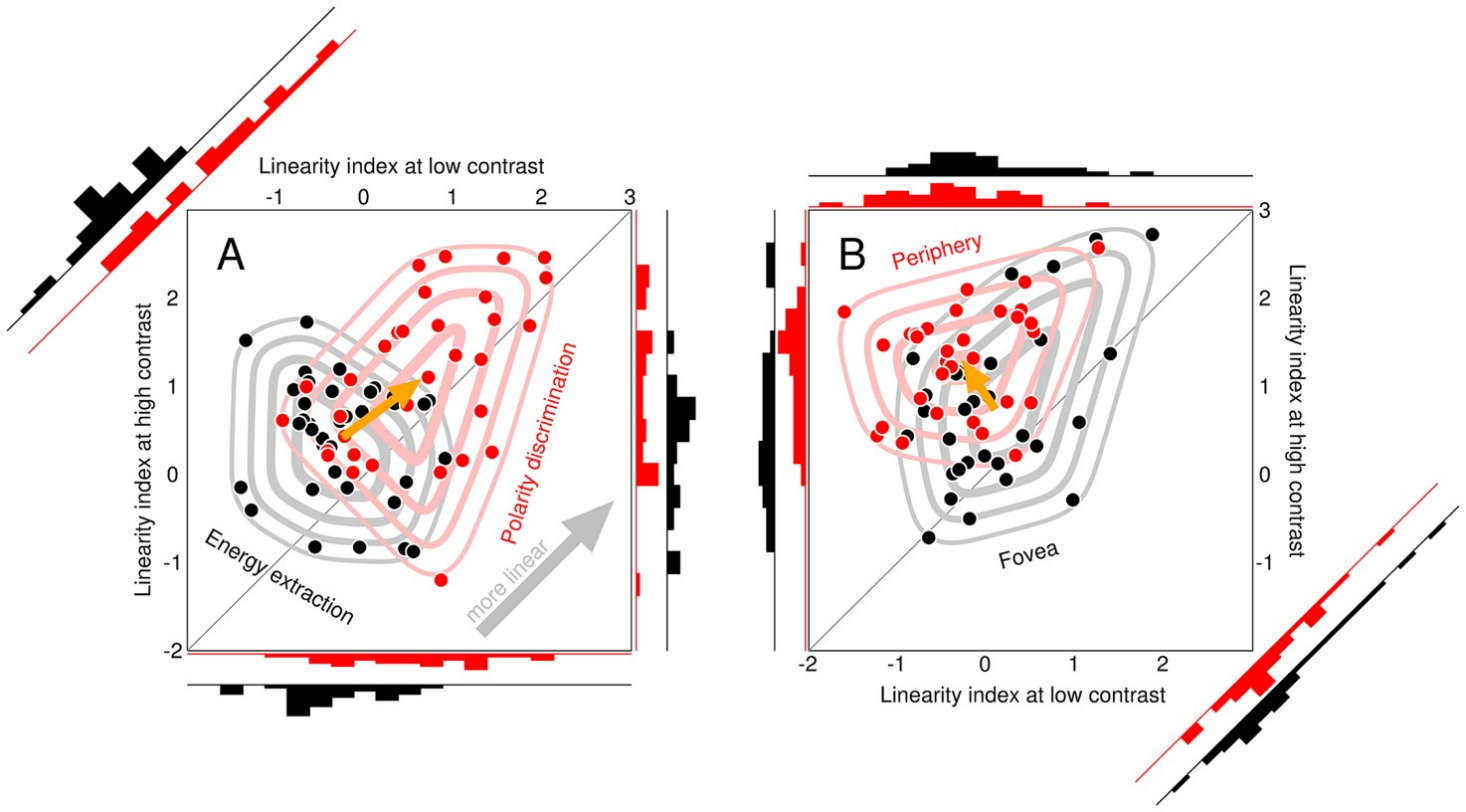
Task-induced steering of system linearity. Symbols plot linearity index (see Methods) at two higher (y axis) versus two lower (x axis) contrast levels separately for each observer and SNR value, from energy-extraction (black) and polarity-discrimination (red) tasks in A, and from peripheral (red) versus foveal (black) conditions in B (both conditions involved the same bright-bar detection task). Error bars are omitted to avoid clutter. Contour lines indicate data spread (from thick to thin, lines refer to 4 different percentage heights of 99/95/70/30 along the surface detailed in the caption to Fig 2). Bottom and right (left in B) histograms plot distributions of x and y values; top-left (bottom-right in B) histograms plot distributions for values projected onto positive diagonal line. Orange arrows point from average coordinates of black data cloud to average coordinates of red data cloud. Red cloud is shifted upwards to the *right* of black cloud in A, indicating greater linearity at both high and low contrast. Red cloud is shifted upwards to the *left* of black cloud in B, indicating no change in overall linearity between the two conditions, but a greater effect of contrast in the periphery compared with the fovea.

When comparing these results with those obtained from the previous experiment (energy extraction), we find that observers can be steered towards adopting more linear or nonlinear mechanisms by rendering the signal-to-be-detected invisible to either mode. This ‘steering’ effect is emphasized in Fig 8A, where it is visually rendered in the form of a cloud-shift of linearity indices (plotted on x/y axes for low/high contrast) between the two tasks of energy (black scatter) and polarity discrimination (red scatter) designed to target nonlinear and linear mechanisms respectively (also labelled ‘energy extraction’ and ‘template matching’ above). Although statistically detectable at both low and high contrast (p<10^−3^), it is relatively small: the difference between the mean values projected along the diagonal (black/red histograms at top-left) normalized by their spread (a metric analogous to d′) is ∼1.4, indicative of substantial overlap between the two task distributions (see also histograms at top-left corner). The incomplete separation between the two processing regimes targeted by our task manipulations suggests that, although it is possible for human observers to recalibrate their perceptual operations in response to changing task requirements, this ability is relatively limited. The origin of this limitation is unclear; in Discussion, we speculate that it reflects constraints imposed by the available neural hardware.

As a way of demonstrating that the effect detailed above is specifically driven by changing task requirements rather than other potential changes of stimulus design and/or experimental protocol, Fig 8B plots equivalent data for one and the same task (bright-bar detection) under two different protocols (temporal versus spatial alternative forced choice) at two different locations in the visual field (fovea versus periphery, black and red symbols respectively in Fig 8B). From the standpoint of stimulus presentation, these differences are arguably greater than those introduced by the two different tasks in Fig 8A, yet Fig 8B does not demonstrate any shift in linear behaviour (there is no transition along the positive diagonal). We conclude that the steering effect does not occur aspecifically for any manipulation of our protocols, but is instead driven by the specific task strategies prompted by our designs.

Fig 8B does, however, demonstrate an orthogonal shift in the direction of greater contrast dependence of the linearity index in the periphery compared with the fovea (orange arrow pointing up to the left). This effect may reflect important differences in the implementation/calibration of certain mechanisms (e.g. gain control) between fovea and periphery. Alternatively, it may simply represent the byproduct of known variations in contrast sensitivity across the visual field [85]: we did not rescale our stimuli to counterbalance the decreased resolution of eccentric locations compared with the fovea, so that a given contrast manipulation of our stimulus may correspond to substantially different manipulations between fovea and periphery (although it is potentially relevant in this respect that there was no difference in efficiency). In the section immediately below we expand on results from the foveal condition.

### Foveal detection

Differences between foveal and peripheral processing are well documented [85], in particular with relation to crowding [86] (this phenomenon being predominantly, if not exclusively, restricted to the periphery). It is therefore important to establish whether the effects we have reported so far in the periphery also generalize to the fovea. They do. Except for the expected overall sharper tuning in the fovea (S6 Fig), all other characteristics are retained (open symbols in Fig 6A); of particular interest is the unequivocal dependence of internal noise on contrast (all red open symbols fall below the horizontal dashed 0 line).

The above result generalizes the contrast-dependent effects across the visual field, and also with relation to the manner in which the two competing stimuli are presented: in the previous experiments, signal+noise and noise-only stimuli were always presented simultaneously to opposite sides of fixation (spatial two alternative forced choice, 2AFC); this mode of presentation is not applicable to the foveal experiments, for which we presented the two stimuli in temporal succession (two interval forced choice, 2IFC). We demonstrate that, despite the potentially different involvement of cognitive factors such as memory and/or expectation between 2AFC and 2IFC protocols [87], these factors play no role in relation to the focus of this study. This finding corroborates the results obtained with the first-trial-only analysis (Fig 5B).

### Off-the-shelf computational models cannot account for the experimental results

**Fig 9:**
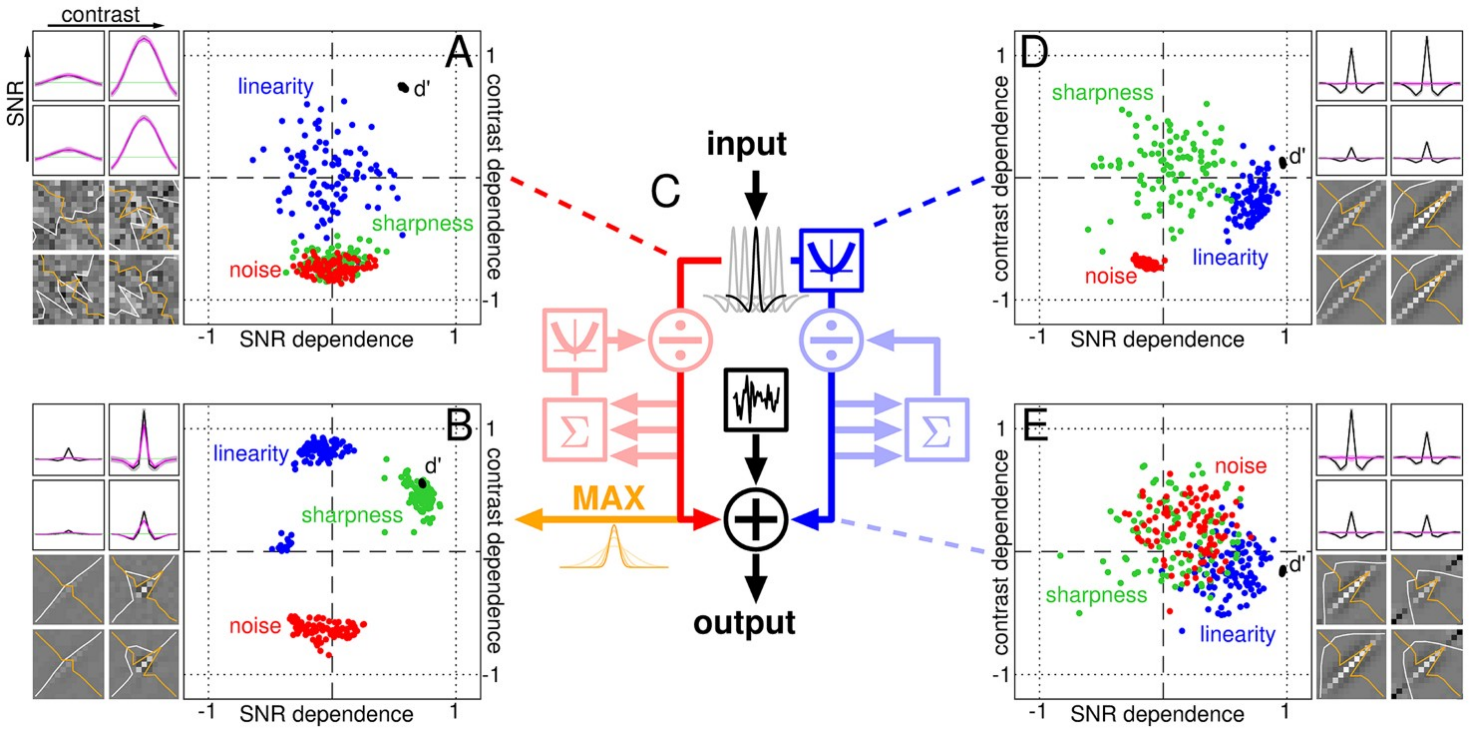
Canonical model components cannot capture the experimental trends. A is plotted to the conventions of Fig 2L; each dot refers to one of 100 simulations (6000 trials per simulation). The four smaller panels to the top-left of A plot target-present/target-absent first-order descriptors to the conventions of Fig 3A, but only for lowest/highest SNR/contrast levels; the four smaller panels to the bottom-left of A plot second-order descriptors to the conventions of Fig 4A. B,D-E are plotted to similar conventions. A refers to a template-matcher model (dark-red branch in C), B to a MAX model (orange branch), D to an energy model (dark-blue branch), E to a divisive normalization model (entire blue branch). Light-red elements in C denote a gain-control circuit acting on the linear branch. Along the nonlinear branch (blue), squaring (parabola within square icon) is applied to each value of the multivariate output from the convolutional later *before* the weighted sum (circled + sign) that leads to the decisional transducer; in contrast, along the linear branch (red) squaring is applied within the gain-control circuit to the sum (∑ sign) of the values returned by the convolutional layer (see Methods for details on model implementation).

The most common model in visual neuroscience (often adopted as simplified implementation of a simple cell [58]) is the linear-nonlinear (LN) cascade (linear filter followed by static nonlinearity), which we implement as a Gaussian-weighted convolution with a bank of linear filters (Mexican-hat shaped traces in Fig 9C) followed by the binary decisional transducer (see Methods). The linearity metric (blue symbols in Fig 9A) correctly demonstrates that the LN model cannot capture the contrast-dependent linear→nonlinear transition. This is true irrespective of parameterization (i.e. specific choice of front-end filter shape, weighting function, internal noise intensity).

The next most popular model in visual psychophysics is the MAX uncertatinty model [30]. In its simplest implementation, this model involves taking the maximum output from the bank of linear filters detailed above. When the ‘attentional window’ (weighting function across filters) is fixed, this model fails miserably. We have therefore implemented a variable attentional window that becomes broader at lower contrasts (orange traces in Fig 9C). This implementation simulates a scenario in which observers are more confident about the expected spatial location of the target signal when contrast is high (narrow attentional window), and are less certain about target location when contrast is low (broader window). It is unsatisfactory for two reasons. First, because it involves an external arbitrary adjustment of model parameters that tracks a property of the stimulus (contrast) without explicitly computing it and incorporating it into the mechanics of the model, however it is the only implementation that allows the MAX model to produce results that are at least marginally compatible with the empirical observations. Second, because the above scenario is highly unlikely given that target position was explicitly indicated by continuous markers of fixed 100% contrast abutting the stimulus (see Methods), so that in fact observers’ confidence about expected target location did not depend on stimulus contrast.

As shown in Fig 9B, the MAX model unequivocally fails to capture the observed effect of contrast on spatial tuning: this characteristic becomes sharper at higher contrast (green data points fall above horizontal dashed line), the opposite of what we observe experimentally. More generally, based on theoretical expectations about the structure of tuning estimates returned by the MAX model [20, 88], sharpness may be enhanced or left unaffected by increasing contrast, but not reduced. Under MAX, target-absent descriptors return the cross-correlation between the front-end convolutional filter and the weighting function [20, 88], while target-present descriptors approximately return the autocorrelation of the convolutional filter [20] (see Methods). For any plausible choice of filter shape and weighting profile, the above implies sharp target-present descriptors and broad target-absent descriptors, similar to what we observe at low contrast. The only strategy for ‘linearizing’ MAX behaviour at high contrast is to narrow the weighting profile to the extent that it only reads out of one channel, reverting to a template matcher. For such a narrow weighting profile, however, first-order descriptors are necessarily sharp (they return the front-end filter), contrary to the empirical observations.

We can further rule out MAX models based on detailed consideration of second-order descriptors. Theoretical expectations about the structure of these descriptors (also confirmed by simulations) impose a strong constraint on the diagonal region: it cannot take negative values under plausible implementations of MAX [20, 24] (see Methods). Previous measurements under conditions that would customarily be modelled using MAX have instead demonstrated unequivocal departures from this prediction, with clear negative modulations along the diagonal region of second-order descriptors [20]. Our additional results from the foveal condition confirm this departure and the associated inapplicability of MAX (S2 Fig). We do not observe negative diagonal modulations in the peripheral condition (Fig 4A); this is expected for broader weighting functions [20] akin to those that must be operating in the periphery [85].

Finally, we consider a barebone energy model and a canonical gain-control circuit, two well-trodden strategies for capturing both neuronal and psychophysical data [48, 89, 90] (these models are often adopted as simplified implementations of a complex cell). A pure energy (quadratic) operator is bound to failure, because it is theoretically expected to produce no modulation for target-absent descriptors (magenta traces within subpanels in Fig 9D; see also Methods). The addition of a gain-control circuit is unable to rectify this conspicuous discrepancy with the data (magenta traces within subpanels in Fig 9E), however it is useful to consider this additional component for two reasons: because it plays an important role in subsequent simulations (see below), and because it implements the classic nonlinear-transduction model often adopted to explain established results in human pattern vision (e.g. dipper effect [60]). This model is clearly inadequate as an explanatory framework for the effects reported here.

Regardless of specific parameterizations, each branch considered above (linear or nonlinear) of the two-branch model in Fig 9C is structurally incapable of capturing the empirical results due to one fundamental reason: the presence of two qualitatively different operating regimes within the human data. Fig 3E demonstrates a clear-cut transition from a highly nonlinear (complex-cell-like) to a highly linear (simple-cell-like) behaviour (red arrow). The models just considered can reproduce one or the other, not both. Our extensive attempts at identifying additional elements (e.g. gain control) that would enable one or the other branch to demonstrate a similar transition were unsuccessful. For this reason, we resort below to a combination of the two branches.

### A hybrid model is satisfactory for qualitative purposes

**Fig 10:**
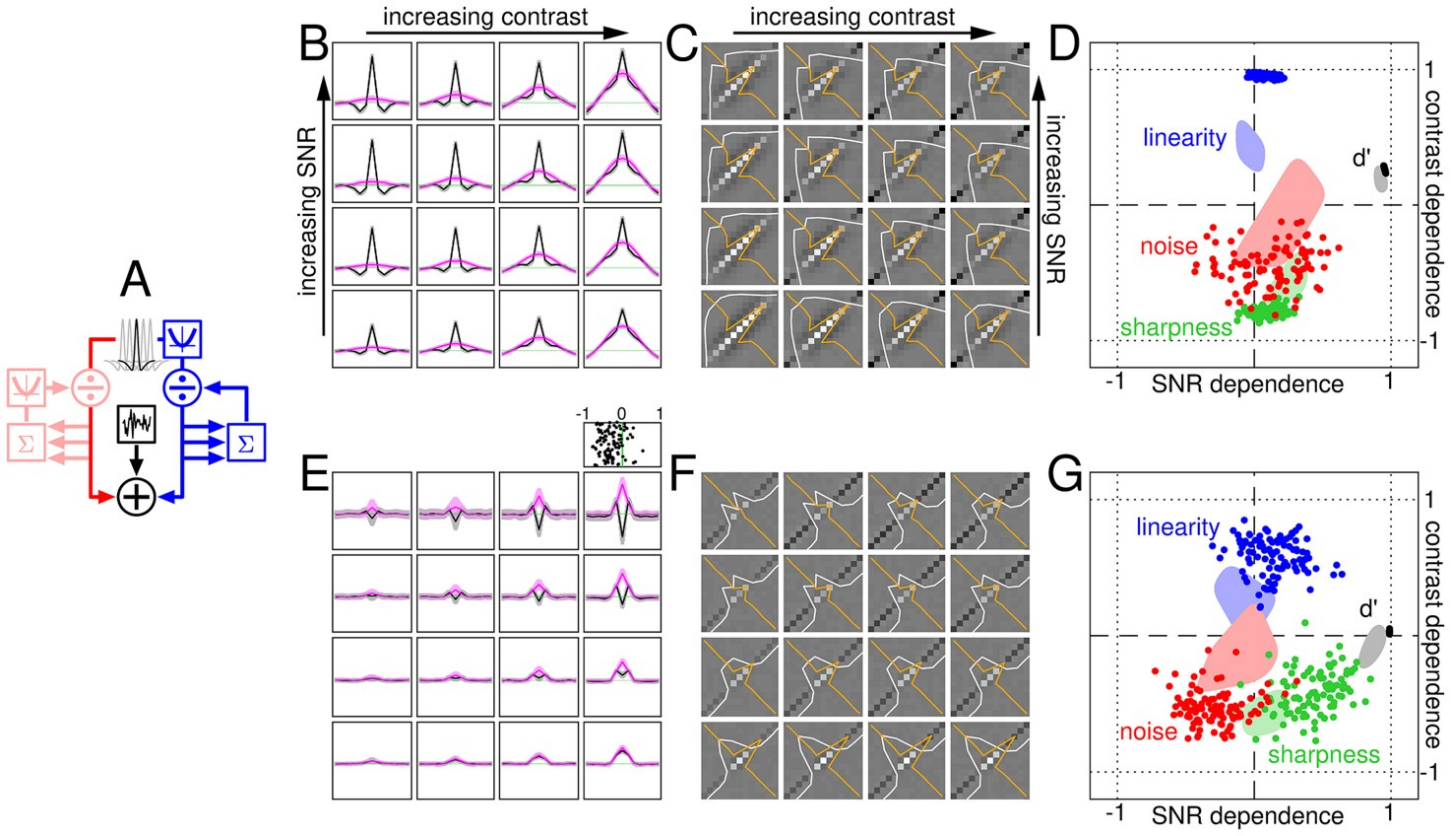
Hybrid two-branch model captures main empirical trends. A-D plot results for a hybrid linear/nonlinear model (dark-red/blue branches in A) where the linear branch is not gain-controlled (light-red component in A is not active); E-G plot results for gain-controlled linear branch (light-red component is active). B is plotted to the conventions of Fig 3A and 10E to the conventions of Fig 7A (each dot in correlation plot at top-right refers to an individual simulation); C,F to the conventions of Fig 4A; D,G to the conventions of Fig 2L. Shaded regions show spread of experimental data for bright-bar detection task (D) and energy-extraction task (G; see caption to Fig 2 for description of how spread is visualized).

Because no single elementary component from canonical off-the-shelf circuits is able to account for basic features of our measurements (see above), we proceed to combine two such components. When linear (red branch in Fig 9C) and energy (blue) operators are combined, the simulated results are remarkably similar to those observed experimentally (compare Fig 10B with Fig 3A). At least qualitatively, the summary metrics generated by this model match those measured from human observers (compare dot clusters with shaded regions in Fig 10D): although the overlap is not quantitatively adequate, the sign of all contrast-dependent effects is correctly reproduced by the model (i.e. whenever a given shaded region lies below/above the horizontal dashed line in Fig 10D, so does the corresponding dot cluster).

The dual-component architecture of this model is responsible for the contrast-dependent shift from nonlinear to linear characteristics: when stimulus contrast is raised, the gain-control circuit greatly reduces the response generated by the energy operator, allowing the linear component to dominate the final output. Although the model reproduces contrast-dependence of internal noise when this quantity is assessed in the form of response inconsistency using our experimental methods (red in Fig 10D), the source of internal noise within the model itself is *fixed* (see Methods). We have also studied the impact of noise sources with varying intensity dictated by stimulus-driven responsivity (often termed ‘multiplicative’ noise in the literature [68]), as well as the differential impact of early versus late sources (see Methods). Modifying noise structure in these ways does not account for our empirical observations (S3 Fig).

To simulate the mixed-polarity results (energy extraction task), we insert an additional gain-control element into the linear subunit (light-red portion of circuit in Fig 10A) to allow for a silencing mechanism of this component; this re-balancing strategy in favour of the energy operator is sensible within the context of the mixed-polarity stimulus/task design. Although model simulations do not perfectly match the human data, they correctly capture the direction of all contrast-dependent effects, including the lack of such effects on sensitivity (black data points in Fig 10G). More strikingly, the simple modification afforded by the additional gain-control element is able to reproduce the puzzling anti-correlation observed at high contrast/SNR for target-present versus target-absent descriptors (see top-right sub-panel in Fig 10E and scatter plot immediately above it; compare with Fig 7A).

**Fig 11:**
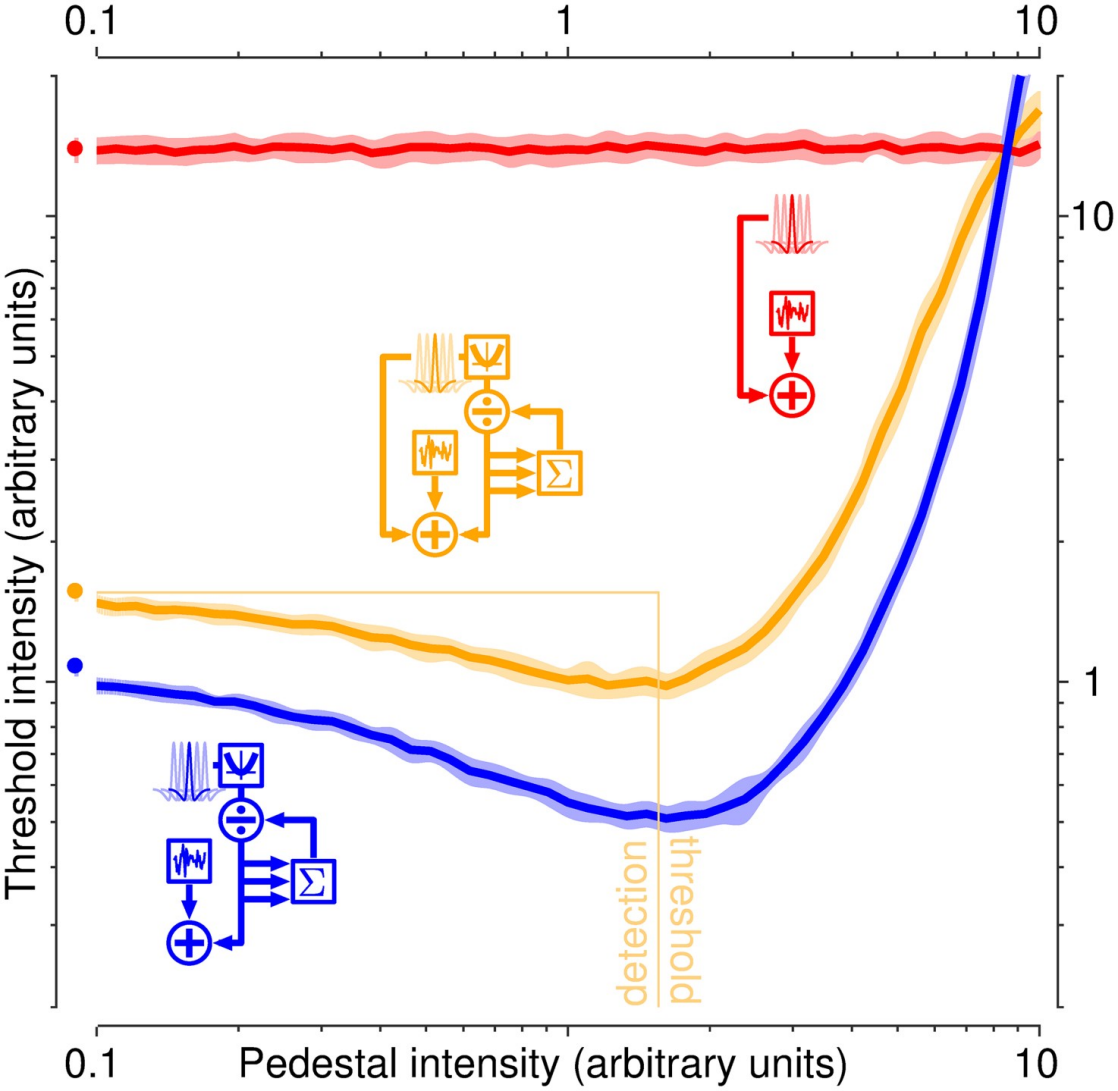
Hybrid model captures the dipper effect. Results from simulations of a contrast discrimination task in the presence of added pedestal contrast (see Methods). Hybrid model (orange) produces the typical threshold-vs-contrast (TvC) dipper function with trough in the vicinity of detection threshold value. The linear branch (red) on its own fails to reproduce the classic dipper characteristic; the nonlinear branch (blue) produces a dipper, albeit slightly shifted to the right of its detection threshold and with an exceedingly steep ‘handle’. Shaded regions show ±1 SD across simulations.

In order for the proposed model to be taken seriously, it must not only explain the empirical findings reported here but also well-known facts from consolidated literature on contrast coding. Perhaps the most established non-trivial result in this area is the ‘dipper’ function, defining the relationship between pedestal contrast and added just-noticeable modulation (the latter being measured as contrast threshold [60]). Without any parameter adjustment, our model is able to capture this classic effect: not only the dipper itself, but its location along the x axis matching detection threshold (orange trace in Fig 11; see Methods). This result is expected due to the presence of the gain-controlled nonlinear branch in the model (blue trace), however the addition of the linear branch brings the slope of the ‘handle’ (rising segment) closer to the typical value found in experiments [60] (∼1).

We offer the above computational account with the understanding that we do not intend for this to be the only account possible, nor for it to capture absolutely every detail of our complex dataset. It is conceivable that simpler computational schemes may achieve similar (if not better) consistency with data. Our primary objective is to show that it is possible to explain our results, at least qualitatively, using models that do not require any external adjustments. It is not necessary to envisage, for example, that observers adopt different models/parameters depending on stimulus contrast: in our simulations, contrast itself as computed by the model is fed back into the circuit via gain control to shape the final response. In this sense, our model is entirely mechanistic. We have also established that observers are unlikely to engage different strategies at different contrasts, because spatial retuning is observed regardless of expectations about the stimulus contrast/SNR due to appear on the next trial (Fig 5B). Further, we demonstrate in Fig 11 that our model, without any tweaking of the parameterization derived from our experiments, is able to account for the most iconic result in pattern vision.

Notwithstanding the qualitative success of this model, there remain quantitative discrepancies that will require further refinement in future work, ideally constrained by additional data. In particular, we alert readers to certain features of the simulated second-order descriptors (Fig 10C and 10F) that are not always evident at the level of the corresponding empirical estimates (Figs 4A, 7B), such as the specific profile displayed along the diagonal region. These discrepancies may in part be attributable to measurement noise in our data, that may hinder recovery of some of those features; it is unlikely, however, that this explanation applies to *all* discrepant features, highlighting the need for further work if the proposed model is to be used for quantitative purposes in future applications.

### Linear/Nonlinear components share common front-end filtering characteristics

A puzzling feature of the hybrid model detailed above is that, while it is able to reproduce the empirically measured contrast-dependence of tuning sharpness as a consequence of the modelled transition from nonlinear to linear regimes, the underlying filtering stage is identical for the two processing modes: both linear and nonlinear branches feed off the same front-end filtering layer, consisting of a bank of sharp (i.e. finely tuned) Mexican-hat-shaped operators (black/gray profiles near top of Fig 10A). This design feature may incorrectly suggest that the two model components should display similar tuning characteristics.

The linear branch displays broader tuning because the output of the front-end layer is weighted by a coarsely tuned envelope along this branch; the two stages being linear, they are equivalent to a single linear stage with broad tuning. Put another way, the fine tuning instantiated by the front-end filtering stage is blurred by subsequent broad pooling of filter outputs. This blurring effect does not occur along the nonlinear branch because the output generated by front-end filtering is subjected to a nonlinear operation (squaring) before undergoing broad pooling by the weighting function; as such, the two operations of front-end filtering and pooling across filters cannot be subsumed under a common linear operation, and the collective properties of the nonlinear component retain the fine tuning characteristics associated with the front-end stage (in the same way that a complex cell may display fine spatial tuning across a broad spatial extent [91]).

**Fig 12:**
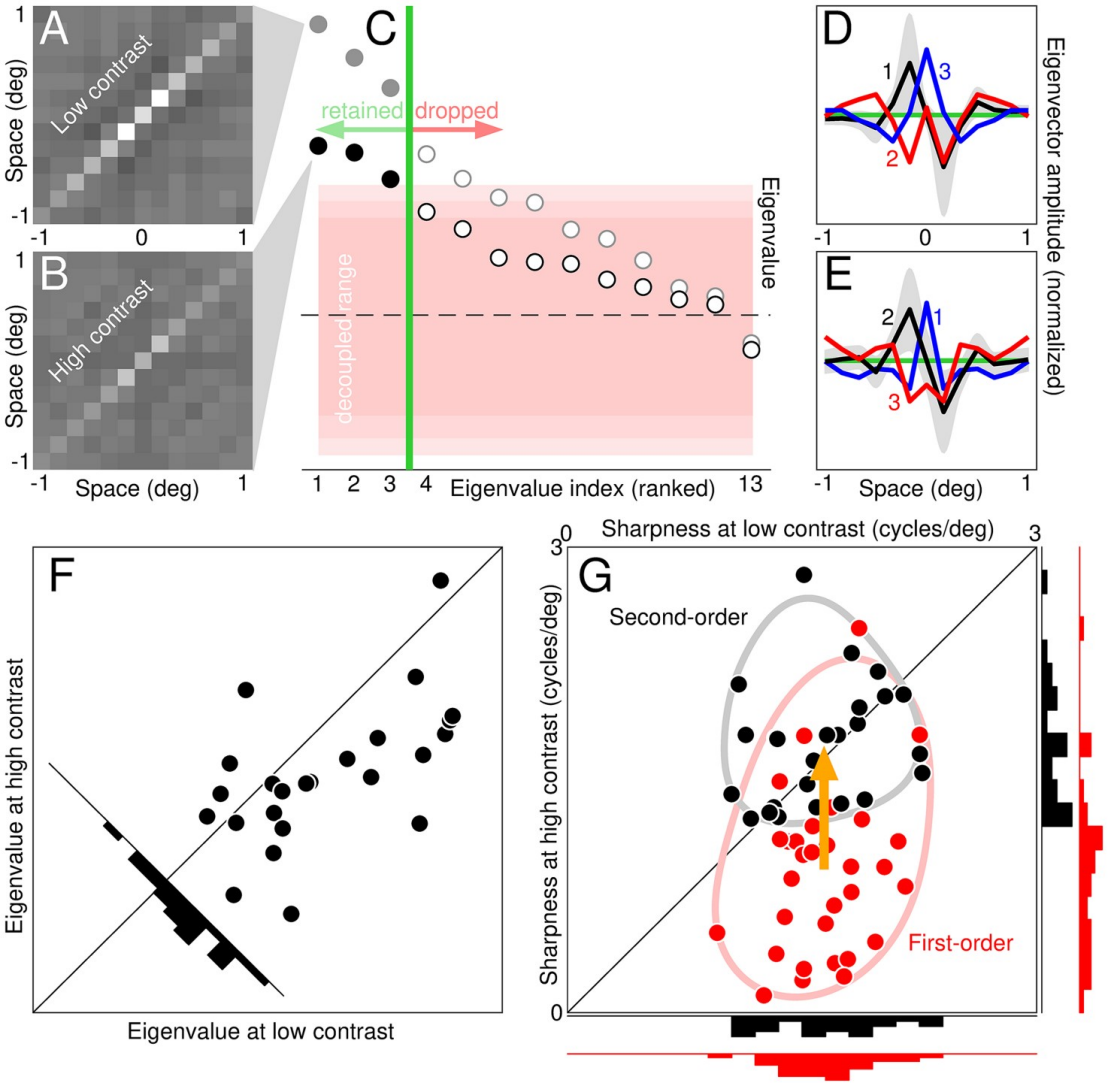
Common front-end convolutional layer at low and high contrast. A shows aggregate (across observers and SNR levels) second-order descriptor at lowest contrast level; B shows same at highest contrast level. Decomposition of the two surfaces produces three significant eigenvalues (solid symbols in C); other values fall within the non-significant range (pink shaded area at ±1× (dark pink), 2× (light) and 3× (lightest) multiples of SD of decoupled distribution; see Methods). D shows eigenvectors corresponding to significant eigenvalues (numeric labels refer to eigenvalue rank) at lowest contrast level; E shows same at highest contrast level. Gray shaded region shows ±1 SEM around black traces; SEM spread is omitted for red/blue traces to avoid clutter, but its magnitude is similar to black traces. F plots significant (1-3) eigenvalues for individual observers at high (y axis) versus low contrast (x axis). Histogram shows distribution for values projected onto negative diagonal. G plots sharpness at high (y axis) versus low contrast (x axis) computed from first-order descriptors (red) separately for each observer and SNR level; contour line indicates data spread (defined in caption to Fig 2). Black symbols were computed from the three significant eigenvectors returned by decomposition of second-order descriptors (averaged across SNR levels), plotted separately for each observer. Orange arrow points from mean coordinates of red cluster to mean coordinates of black cluster. Bottom/right histograms plot distributions for x/y values. Error bars are omitted from F-G to avoid clutter.

We now ask whether we can validate this model feature with data: is it possible to expose signatures of filtering characteristics that are common to both low and high contrast regimes from the empirically measured perceptual process? From the analysis presented so far, those characteristics are markedly different: as extensively detailed above and as further summarized in Fig 12G, first-order sharpness is greater at low (plotted on x axis) versus high (y axis) contrast (red data points fall below diagonal unity line). Although this marked difference can be modelled using a common front-end filtering bank (see paragraph above), such evidence for a common filtering stage remains indirect as it is based on articulated computational models.

To achieve the above-stated goal, we resort to powerful data reduction techniques. In particular, second-order descriptors (Fig 12A and 12B) can be reduced to a compact description via eigenvalue decomposition [39, 46] (see Methods). The resulting significant eigenvectors are easy to visualize (Fig 12D and 12E); if the perceptual operator is well approximated by a bank of linear filters followed by squaring (blue branch in Fig 10A), the eigenvectors recover the characteristics of the underlying filters [39]. Fig 12G shows that eigenvector sharpness is similar at low and high contrast (black data), consistent with the model architecture in Fig 10A. The corresponding eigenvalues, however, are larger at lower contrast (data points fall below diagonal unity line in Fig 12F and black symbols fall below gray symbols in Fig 12C), consistent with a greater role for the nonlinear component.

Incidentally, the result of matched sharpness from second-order descriptors argues against the possibility that the observed difference from first-order descriptors is due to noisier estimates at low contrast (see Methods), because such greater noisiness should apply to estimates from second-order descriptors too. It is also noteworthy that there are two distinct ways in which sharpness may shift from first-order estimates, scattered *away* from the unity line in Fig 12G, to second-order estimates, scattered *around* the unity line. The shift may occur along the x axis: the low-contrast descriptor becomes less sharp, to match reduced sharpness at high contrast; or it may occur along the y axis: the high-contrast descriptor becomes sharper, to match enhanced sharpness at low contrast. Only the latter scenario is supported by data (see upward-pointing orange arrow in Fig 12G); this is also the scenario expected for the modelling architecture in Fig 10A.

## Discussion

### Relations to prior literature

Two previous studies have reported perceptual sharpening at low contrast. In the pioneering study by Fiorentini and Mazzantini [71], the authors speculate that this effect may reflect linear processing at low contrast and nonlinear processing at high contrast (see also [92, 93]); our direct measurements of linearity/nonlinearity expose the opposite trend. In the more recent study by Abbey and Eckstein [75], the authors adjust stimulus SNR to target similar performance across contrast levels; contrast and SNR therefore co-vary in their experiments, raising the possibility that the measured effect on tuning may be produced by changes in SNR, not contrast. We can exclude any role for sensitivity/efficiency/SNR in shaping spatial tuning because we varied stimulus SNR (and sensitivity/efficiency along with it) as a control manipulation. We also measured internal noise, thus gaining access to greater detail of characterization than afforded by the two studies just considered. It is remarkable that, despite the numerous differences between those two studies and our own, all measurements expose sharper tuning at low contrast. Most remarkably, our stimulus duration (50 ms) is 1 order of magnitude shorter than adopted by Abbey and Eckstein [75] (500 ms), and the earlier study [71] allowed open-ended foveal inspection time, implying that the tuning effects reported in this article generalize to much longer presentation times.

We measure internal noise using the double-pass technique, arguably the most direct method for assessing behavioural instrinsic variability [27]. The contrast-dependence of this quantity as documented here is at odds with theoretical work on this topic. Some authors have hypothesized that internal noise may be higher at lower contrast due to a varying noise source [94]; our results indicate instead that the noise source itself is likely fixed (see also [95]), but generates contrast-dependent response variability as a consequence of activity balance within the circuit (red symbols in Fig 10D). Recent theoretical work has hypothesized that internal noise may reflect sizeable mismatch between template mechanisms and input signals [96]. Our results are inconsistent with this interpretation, as best exemplified by the lack of measurable changes in sensitivity with increasing contrast in the energy-extraction task (black symbols in Fig 7C), despite a concomitant decrease in internal noise. This result implies that the discriminatory power of the sensory operator before the addition of internal noise must be greater at lower contrast or, equivalently, that the processing machinery is better matched to the target signal when internal noise is larger, opposite to what is predicted by current theoretical accounts [96].

There are striking similarities between some of the effects we measure here and those reported for single neurons in early visual cortex. In a series of experiments documenting perhaps the most detailed characterization of relevant effects available from the literature, Fournier and colleagues [39, 61] derived both first-order and second-order descriptors for visual neurons in cat V1. Through detailed comparison of the experimental characterization and predictions from physiologically plausible computational models, they pin-pointed a model architecture (termed GC3 in [61]) that is nearly identical to the two-branch structure constrained by our own data. This convergence of data-driven computational efforts may be coincidental, or it may expose an important connection between neural circuits and behaviour.

Although not directly relevant to our work, it is also noteworthy that empirical trends analogous to those reported here have been observed in the auditory domain, and that similar modelling schemes are generally considered as suitable accounts of those trends. More specifically, frequency tuning of both auditory neurons [97] and human listeners [98] shifts from bandpass to lowpass with increasing sound level. This transition is well captured by a hybrid model with two branches [62], one linear and the other nonlinear (similar to the model structure depicted in Fig 10A). The transition occurs due to the dominance of the nonlinear branch at low levels and the take-over of the linear branch at higher levels, just as we propose here for increasing contrast. Despite the important material and conceptual differences between the two phenomena, including the dubious connection between visual contrast and sound level, it is nevertheless interesting that biological sensory processing appears to exploit similar computational motifs for handling different types of signals.

### Theoretical implications

Our results are directly relevant to pattern vision, but their significance extends to the role played by theory in informing our understanding of human sensory processing. Nearly every effect we report here is contrary to what is expected from current theoretical accounts. If we adopt a normative approach, the benchmark is represented by the ideal observer [19]. This theoretical construct makes straightforward predictions (see Methods): for all experiments bar the mixed-polarity task, first-order descriptors should be scaled images of the target signal [23, 29] and second-order descriptors should be featureless [24]. Human observers clearly depart from this prediction, forcing consideration of alternative theoretical/computational frameworks.

We consider two distinct approaches: we either focus on retaining optimality, but we define ‘optimal’ with reference to more general conditions than those specified by our stimulus/task design; alternatively, we consider canonical circuits that dominate current computational literature in connection with contrast coding. If we take the former approach, lowering contrast against fixed intrinsic variability amounts to decreasing the SNR of the internal representation. From a general theoretical standpoint, the optimal strategy for the sensor is to switch from redundancy reduction to averaging [18]; within the context of pattern vision, this transition involves a highpass-to-lowpass recalibration. Effects of this kind have been observed in the temporal domain for some neuronal types [99] and human vision [100, 101]; in the spatial domain, they may be connected with contrast-driven alterations of receptive-field size [102, 103] and perceptual summation [104, 105], although explicit neuronal measurements of spatial frequency selectivity have not delivered a picture that is entirely consistent with accounts of this kind [106]. We observe the opposite effect, calling into question the suitability of optimality as the most effective way of approaching these issues.

We can take the alternative view that the system is primarily constrained by whatever circuitry is available in cortex (a view to which we subscribe here, although combined with the adoption of new models). This is not to say that optimality becomes an irrelevant factor: cortical circuits have evolved to deliver successful behaviour, and it therefore follows that they must be at least ‘good’ if not ‘optimal’. When good is good enough, however, remains unclear. Some authors have proposed that optimality must be traded against the cost of implementing it with neural hardware: cheap simple circuits may deliver adequate performance for survival without the need to represent the optimal statistical solution explicitly [13], nor achieve the level of performance prescribed by that solution [12]. Among canonical circuits, the most successful operator for capturing the impact of contrast on pattern vision is the gain-control normalization model [90]. This model cannot account for conspicuous features of our dataset (Fig 9E). The notion of divisive normalization *per se* is therefore inadequate as an interpretational tool for our results (although it represents an important component of more articulated models that are able to capture the experimental observations, see Fig 10).

### Constrained access to a canonical format

Our favoured interpretation is, in many ways, simpler. Visual cortex cannot display an infinite repertoire of computational motifs: despite its staggering complexity and flexibility, its operations must be limited by the characteristics of the hardware that underlies those operations. Based on current knowledge of cortical circuits, it is our understanding that image content is initially submitted to a limited set of canonical computations [107]. Once information is formatted in this way, it is not possible to ‘undo’ those computations at will: any subsequent unpacking of the early image representation must be constrained by the formatting code initially imposed on the visual stimulus. We speculate that the properties and rules implemented by the early coding mechanisms are reflected in the trends exposed by our experiments, such as the contrast dependence of spatial tuning and internal noise.

Viewed from this perspective, those trends are not necessarily expected to follow specific theoretical predictions: their basic behaviour is constrained by the mechanisms available to do the coding, whether those mechanisms are optimal or not for the problem at hand. Optimality does play a role in the sense that it is possible to ‘steer’ perceptual computations towards different strategies depending on the suitability of those strategies for the assigned task (Fig 8); this steering process, however, is limited in scope. More specifically, its limitation stems from the finite repertoire of perceptual modes that can be engaged for any given task, and from the bounded affordability of differential engagement for those modes: the available perceptual strategies may be restricted to coarse approximations of desired computations such as energy extraction and template matching, rather than exact implementations of those operations; further, it may not be possible to completely shut down one strategy and only rely on the other, even though it may be desirable from the standpoint of optimal behaviour.

There is another possibility of course, which is that we currently do not understand the theory behind these issues well enough to explain why the system displays the effects reported here, and that future theoretical accounts of how pattern vision should operate in relation to natural signals (e.g. [8, 10]) may predict the exact properties we measure in human observers. Our results do not argue against this possibility; to the contrary, they prompt and constrain further theoretical investigations aimed at achieving this objective, hoping that it can indeed be achieved. Until such satisfactory accounts are developed, however, our empirical results send a warning signal that there may be important unresolved issues with the way we currently approach sensory processing from a theoretical perspective, and that there may be a lot more experimental facts we need to gather before we begin to understand even the most basic operations that are carried out by human vision [11].

## Supporting information

S1 Fig Internal noise estimates are not substantially affected by response bias. A plots response bias distribution across our entire dataset for a total of 592 independent estimates (one from each observer, task, SNR/contrast pairing). Bias is in d′ units [29]. B plots the 439 internal noise estimates that fall within the plausible range [20] (>1/5 and <5) when corrected (y axis) and not corrected for response bias (x axis). Please refer to Methods for how these quantities were computed.
(TIF)

S2 Fig Second-order diagonal is inconsistent with MAX model. A plots the full second-order descriptor for the foveal condition (see Methods) aggregated across observers. Positive modulations are bright, negative modulations are dark (gray is zero). Red ovals highlight negative modulations within the diagonal region. B plots diagonal profiles separately for each of 8 observers (shading shows ±1 SEM). Different traces have been offset vertically to ease visualization; the 0 point (green) on the y axis is marked separately for each trace. All traces display negative modulations to the sides of the central peak.
(TIF)

S3 Fig Different types of simulated internal noise cannot account for the empirical observations. B-E are plotted to the same conventions of Fig 9A, 9B, 9D and 9E. B-C refer to the energy model without (B, dark-blue branch in A) and with divisive normalization (C, entire blue branch in A). Differently from the related simulations in Fig 9D and 9E, the source of internal noise acts early in the model at the level of the front-end convolutional layer (black square outline in A) rather than the level of the late weighted summation stage (light-gray square outline in A, same as black square outline in Fig 9C). D-E are equivalent to B-C except the early source of internal noise is modulated in a responde-dependent fashion (see Methods). In the latter implementation, the intensity of the internal noise that is added to the output of each filter in the convolutional layer scales positively with said output: if the filter responds more vigorously, it also presents more additive intrinsic variability. This type of variability demonstrates SNR-dependence of the internal noise estimated from response consistency (see red symbols in the scatter plots of D-E), consistent with the notion that increasing stimulus SNR leads to greater responsivity of the filters targeting the stimulus region in the vicinity of the target, therefore resulting in greater intrinstic variability. The SNR-dependent trend, however, is not observed in the human data (Fig 1C).
(TIF)

S4 Fig Results from dark-bar detection experiments match those from bright-bar detection. Plotted to the same conventions of Fig 3. Notice expected sign-inversion of first-order descriptors (A-B).
(TIF)

S5 Fig Results from polarity discrimination experiments, plotted to the same conventions of Fig 3. Notice overall greater degree of linearity (E) than observed for bright-bar detection (Fig 3E).
(TIF)

S6 Fig Results from foveal detection experiments, plotted to the same conventions of Fig 3. Notice overall sharper tuning of first-order descriptors (B-C) than their peripheral counterparts (Fig 3B and 3C).
(TIF)

S1 Data Labelled dataset in Matlab format (*.mat). (MAT)
