# Supplementary figures and images for: The empirical characteristics of human pattern vision defy theoretically-driven expectations
Source: PLoS Comput Biol. 2018 Dec 4;14(12):e1006585. doi: 10.1371/journal.pcbi.1006585 (PMC6294397; doi:10.1371/journal.pcbi.1006585)

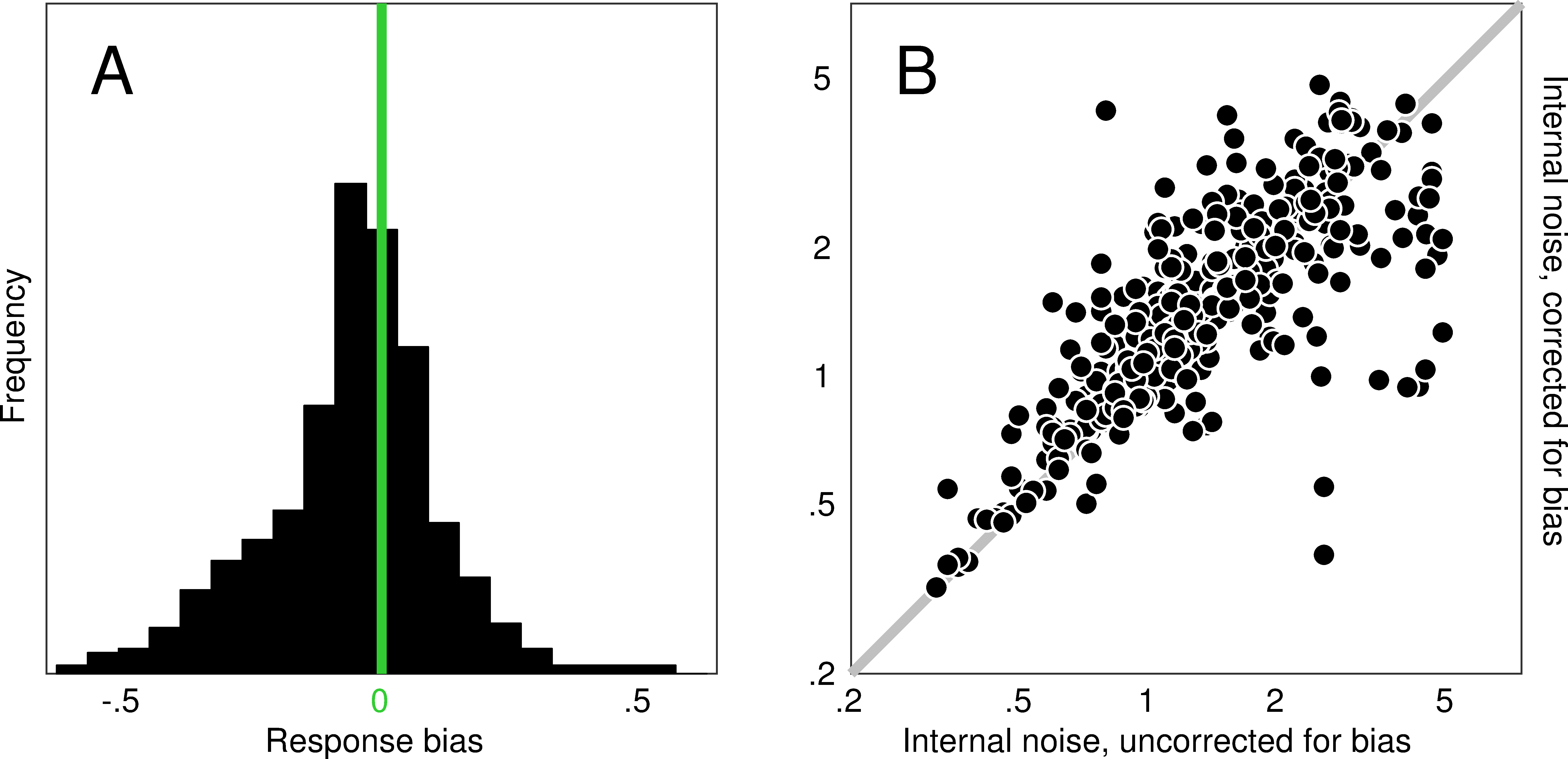

Supplement: S1 Fig — A plots response bias distribution across our entire dataset for a total of 592 independent estimates (one from each observer, task, SNR/contrast pairing). Bias is in d′ units [29]. B plots the 439 internal noise estimates that fall within the plausible range [20] (>1/5 and <5) when corrected (y axis) and not corrected for response bias (x axis). Please refer to Methods for how these quantities were computed. (TIF) [file pcbi.1006585.s001.tif]

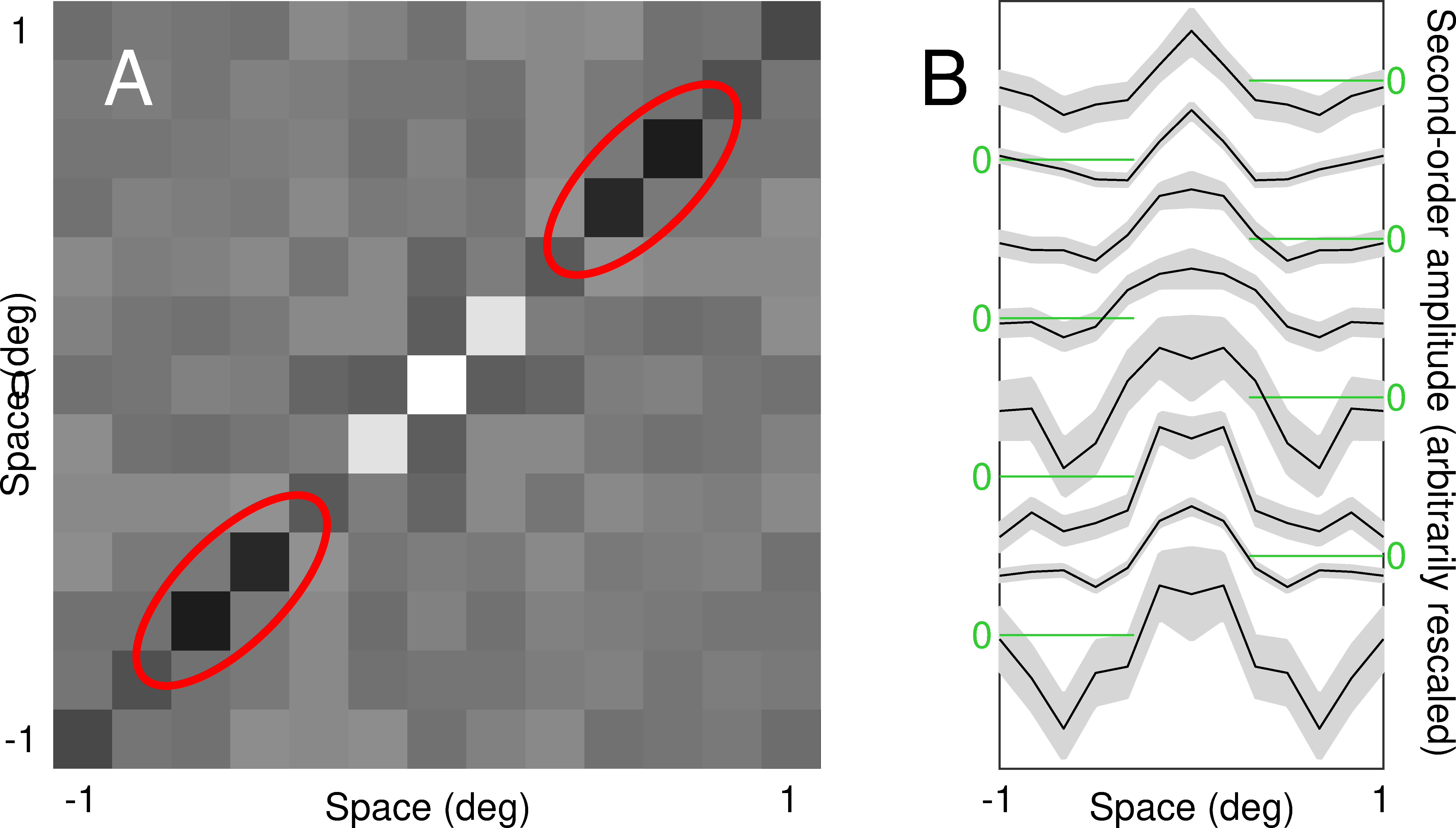

Supplement: S2 Fig — A plots the full second-order descriptor for the foveal condition (see Methods) aggregated across observers. Positive modulations are bright, negative modulations are dark (gray is zero). Red ovals highlight negative modulations within the diagonal region. B plots diagonal profiles separately for each of 8 observers (shading shows ±1 SEM). Different traces have been offset vertically to ease visualization; the 0 point (green) on the y axis is marked separately for each trace. All traces display negative modulations to the sides of the central peak. (TIF) [file pcbi.1006585.s002.tif]

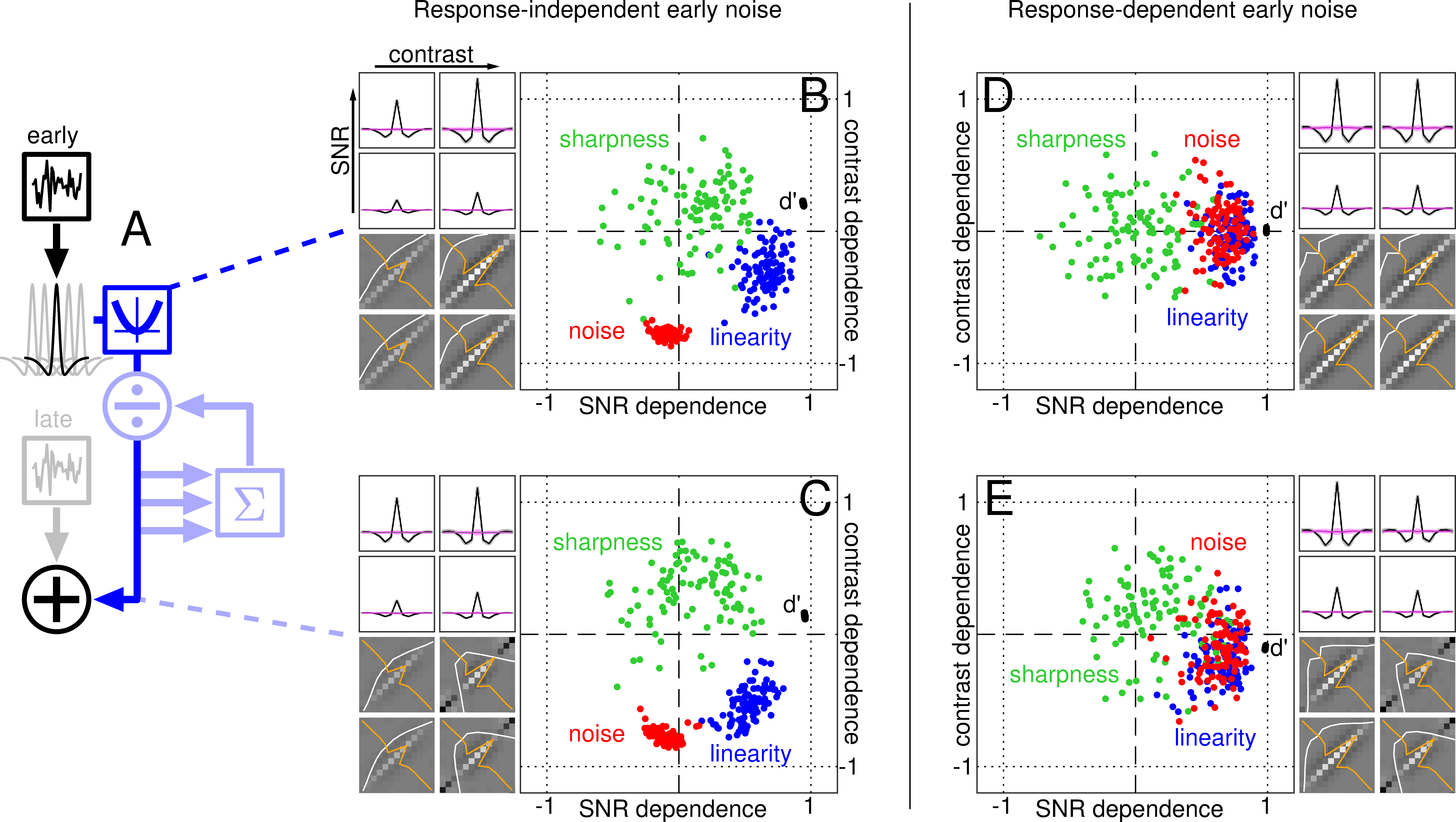

Supplement: S3 Fig — B-E are plotted to the same conventions of Fig 9A, 9B, 9D and 9E. B-C refer to the energy model without (B, dark-blue branch in A) and with divisive normalization (C, entire blue branch in A). Differently from the related simulations in Fig 9D and 9E, the source of internal noise acts early in the model at the level of the front-end convolutional layer (black square outline in A) rather than the level of the late weighted summation stage (light-gray square outline in A, same as black square outline in Fig 9C). D-E are equivalent to B-C except the early source of internal noise is modulated in a responde-dependent fashion (see Methods). In the latter implementation, the intensity of the internal noise that is added to the output of each filter in the convolutional layer scales positively with said output: if the filter responds more vigorously, it also presents more additive intrinsic variability. This type of variability demonstrates SNR-dependence of the internal noise estimated from response consistency (see red symbols in the scatter plots of D-E), consistent with the notion that increasing stimulus SNR leads to greater responsivity of the filters targeting the stimulus region in the vicinity of the target, therefore resulting in greater intrinstic variability. The SNR-dependent trend, however, is not observed in the human data (Fig 1C). (TIF) [file pcbi.1006585.s003.tif]

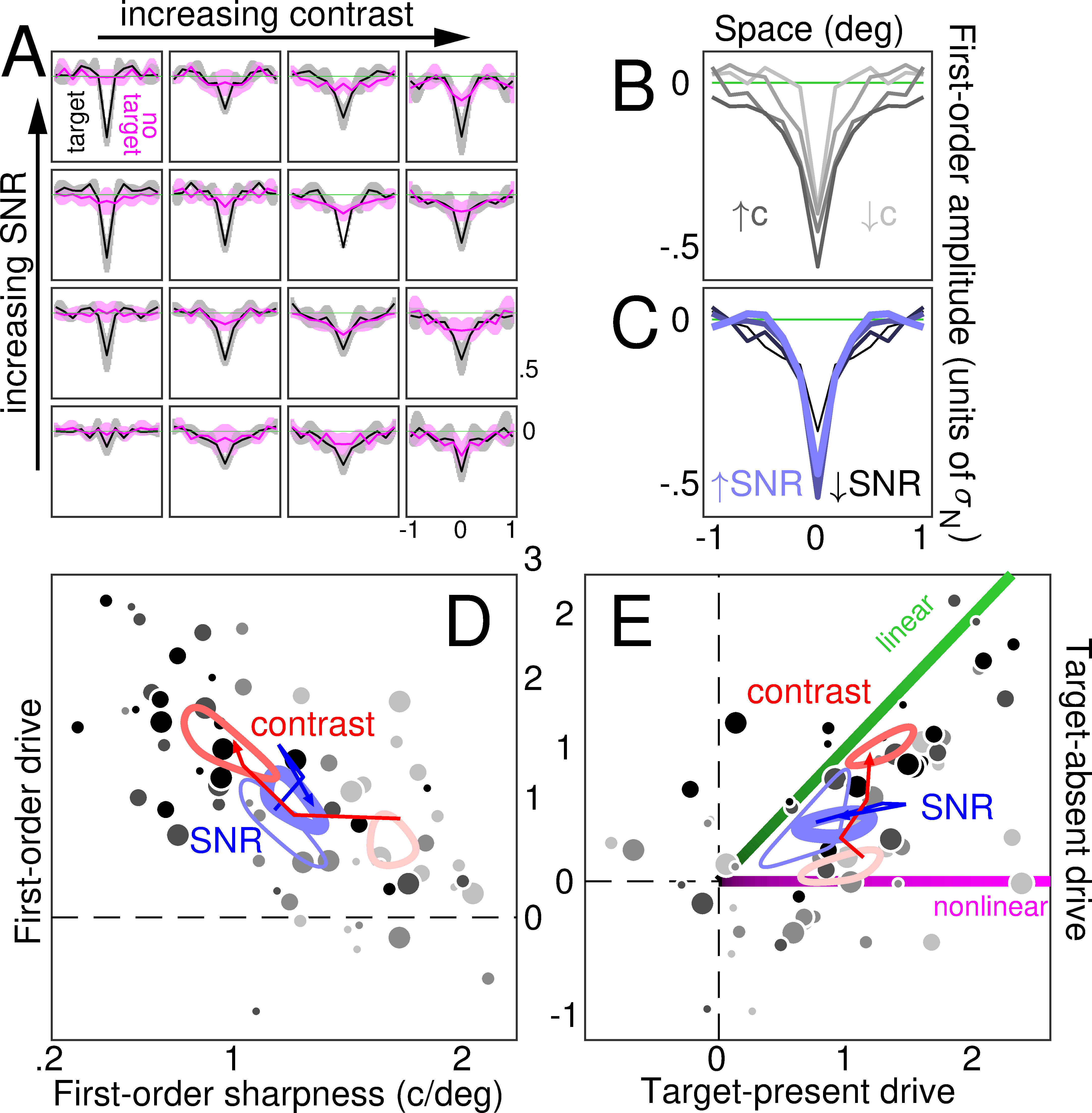

Supplement: S4 Fig — Plotted to the same conventions of Fig 3. Notice expected sign-inversion of first-order descriptors (A-B). (TIF) [file pcbi.1006585.s004.tif]

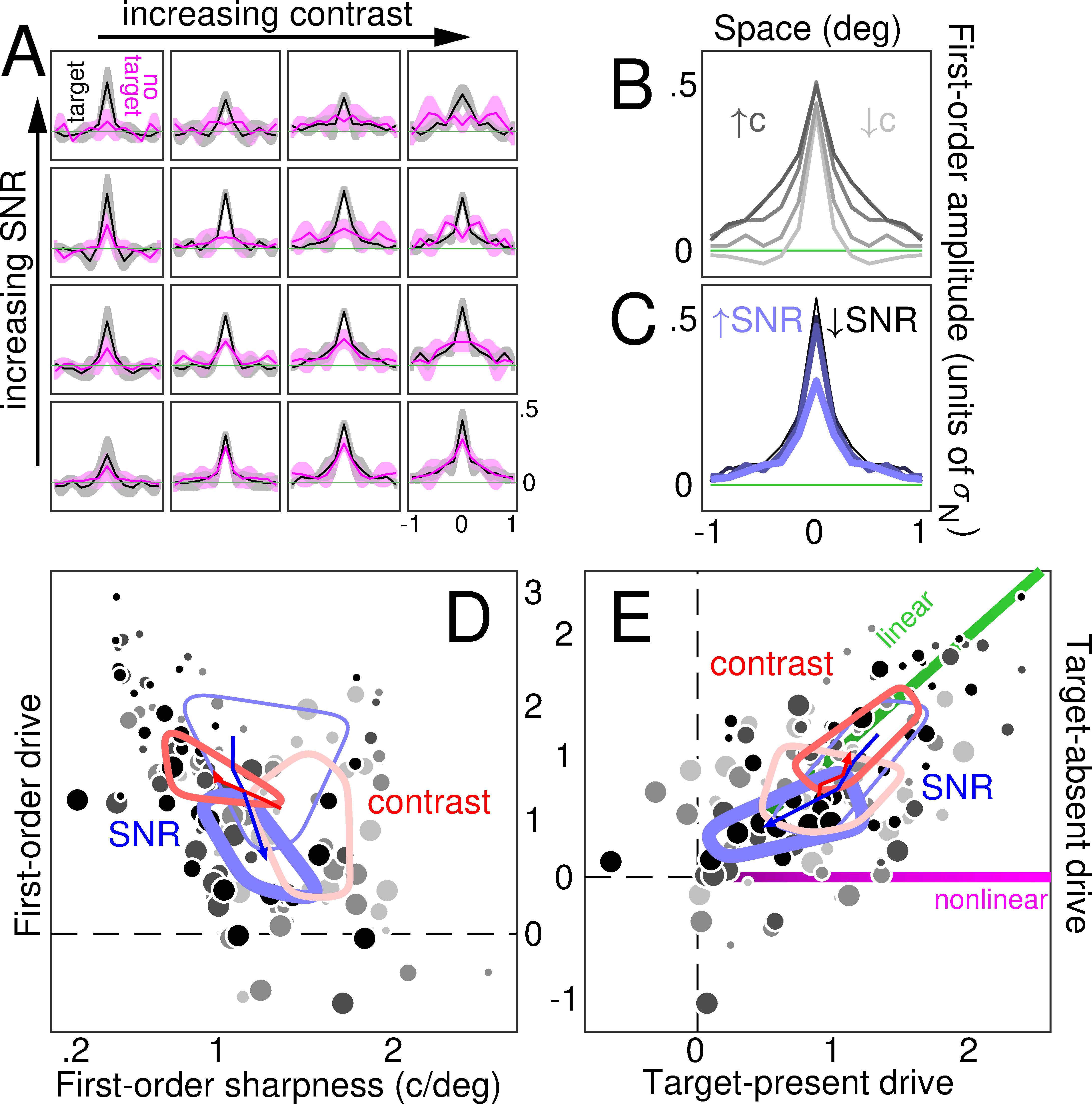

Supplement: S5 Fig — Notice overall greater degree of linearity (E) than observed for bright-bar detection (Fig 3E). (TIF) [file pcbi.1006585.s005.tif]

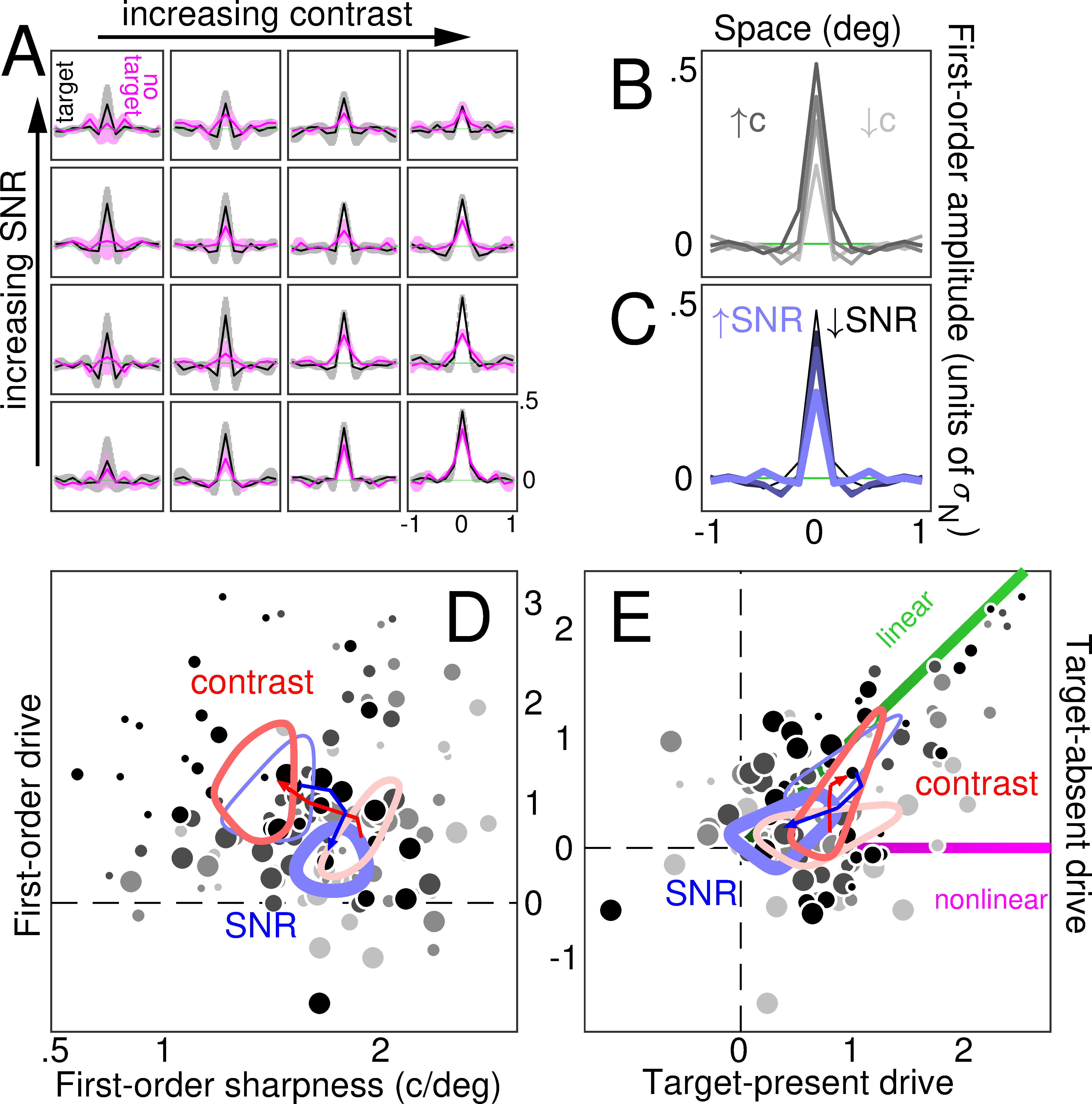

Supplement: S6 Fig — Notice overall sharper tuning of first-order descriptors (B-C) than their peripheral counterparts (Fig 3B and 3C). (TIF) [file pcbi.1006585.s006.tif]
